# Combined expansion and STED microscopy reveals altered fingerprints of postsynaptic nanostructure across brain regions in ASD-related SHANK3-deficiency

**DOI:** 10.1038/s41380-024-02559-9

**Published:** 2024-04-22

**Authors:** Jan Philipp Delling, Helen Friedericke Bauer, Susanne Gerlach-Arbeiter, Michael Schön, Christian Jacob, Jan Wagner, Maria Teresa Pedro, Bernd Knöll, Tobias M. Boeckers

**Affiliations:** 1 Institute of Anatomy and Cell Biology, Ulm University, Ulm, 89081 Germany; 2 Max Planck Institute of Psychiatry, Munich, 80804 Germany; 3 Department of Neurology, Ulm University, Ulm, 89081 Germany; 4 Department of Neurosurgery, Ulm University, Ulm, 89081 Germany; 5 Institute of Neurobiochemistry, Ulm University, Ulm, 89081 Germany; 6 Ulm Site, DZNE, Ulm, 89081 Germany

**Keywords:** Autism spectrum disorders, Neuroscience

## Abstract

Synaptic dysfunction is a key feature of SHANK-associated disorders such as autism spectrum disorder, schizophrenia, and Phelan-McDermid syndrome. Since detailed knowledge of their effect on synaptic nanostructure remains limited, we aimed to investigate such alterations in ex11|SH3 SHANK3-KO mice combining expansion and STED microscopy. This enabled high-resolution imaging of mosaic-like arrangements formed by synaptic proteins in both human and murine brain tissue. We found distinct shape-profiles as fingerprints of the murine postsynaptic scaffold across brain regions and genotypes, as well as alterations in the spatial and molecular organization of subsynaptic domains under SHANK3-deficient conditions. These results provide insights into synaptic nanostructure in situ and advance our understanding of molecular mechanisms underlying synaptic dysfunction in neuropsychiatric disorders.

## Introduction

Optical microscopy has revolutionized the life sciences, enabling numerous breakthroughs in our understanding of physiological and disease-associated biological processes. In particular, super-resolution microscopy (SRM) techniques such as STED [1, 2], SIM [3, 4], STORM [5–7], PALM [8], PAINT [9–11] and others have pushed the resolution limits of fluorescence microscopy and allowed for more detailed investigation of cellular structures. However, these approaches often face challenges when applied to thick tissue samples, limiting their applicability for studying synaptic nanostructure in situ. Expansion Microscopy (ExM) surpasses the resolution limit imposed by the diffraction of light by physically enlarging the sample through embedding in a swellable polyacrylate gel [12]. Due to the innate properties of expanded hydrogels, ExM has proven highly suitable for imaging densely packed tissues like the nervous system, including neuronal synapses [13–17].

The nanostructural organization of synapses is highly complex and finely regulated, with numerous proteins assembled into distinct subsynaptic domains (SSDs) and trans-synaptically aligned nanocolumns, which play key roles in effective neurotransmission and synaptic plasticity [18–24]. Understanding synaptic nanostructure is thus essential for elucidating the mechanisms governing these processes. This knowledge is particularly relevant to neurodevelopmental disorders such as autism spectrum disorder (ASD), that have been linked to genes associated with neuronal synapses [25]. One key molecular player implicated in these disorders is SHANK3, a scaffolding protein enriched at the postsynaptic density (PSD) of excitatory synapses and crucial for synaptic organization and function [26–29]. Genetic alterations affecting *SHANK3* have been identified in patients with ASD, schizophrenia, intellectual disabilities, and Phelan-McDermid syndrome (PMDS) [30–33].

Despite advances in our understanding of SHANK3’s role in neuropsychiatric disorders, detailed knowledge about synaptic nanostructure under SHANK3-deficient conditions remains limited. This is mainly due to the resolution constraints of conventional microscopy and a lack of molecular context provided by electron microscopy. In this study, we aimed to develop an ExM protocol optimized for imaging neuronal synapses in situ using archival murine and human brain tissue while achieving robust compatibility with STED microscopy (ExM-STED). We employed ExM and ExM-STED to investigate the nanostructural organization of synapses across cortical and non-cortical brain regions in wild type (WT) and ex11|SH3 Shank3-knockout (KO) mice [34–36]. Our findings provide novel insights into the consequences of SHANK3-deficiency on subsynaptic organization, advancing our understanding of synaptic dysfunction in neuropsychiatric disorders.

## Materials and methods

### Animals

The homozygous ex11|SH3 *Shank3*-knockout (*Shank3*^−/−^; KO) and wild type (*Shank3*^+/+^, WT) mice used in this study were bred as described previously [34, 36]. All mice were housed under constant temperature (22 ± 1 °C) and humidity (50%), with a 12 h light/dark cycle, and provided with food and water ad libitum. Using heterozygous breedings, we derived WT and KO littermates, which were genotyped by PCR. Mice were grouped as same-sex littermates. For Expansion Microscopy (ExM), six 18-weeks-old mice (3x WT, 3x KO) from a single cohort were used.

### Transcardial perfusion of mice & sectioning

For immunohistochemistry of the brain via ExM, mice were anesthetized and transcardially perfused with 25 mL of ice-cold 1x DPBS −/− (Gibco, 14190250) followed by 50 mL of ice-cold 4% formaldehyde (FA; Sigma, 158127) in 1x DPBS −/− at pH 7.4. Collected tissue was post-fixed in 4% FA in 1x DPBS −/− overnight and cryoprotected with 30% sucrose (Sigma, S0389) in 1x DPBS −/− for approximately 48 h at 4 °C. Finally, samples were snap-frozen in Tissue-Tek O.C.T (Sakura, 4583) and stored at -80 °C until they were serially sectioned into 40 μm thick coronal brain slices using a cryostat (Leica, CM3050 S). Free-floating slices were collected, washed 3x in 1x DPBS −/−, and then stored in 30% ethylene glycol (Carl Roth, 6881.1), 20% glycerol (Sigma, 15523), 50% 1x DPBS −/− (cryoprotectant solution) at −20 °C until further processing.

For genotype and brain region comparisons, slice positions were selected according to the Allen Mouse Brain Atlas at a coronal section position (reference position 190), in which the corpus callosum is connected and the anterior commissure appears as a dot in both hemispheres. At this position, all target regions - striatum (STR), sensory (SENS), and motor cortex (MOT) - were readily dissectable.

### Fixation procedure for human samples

Following epilepsy surgery of the 21-year-old female patient, the supplied resected tissue (origin: temporal pole) was immediately immersion-fixed in a solution according to Tutsch (anatomy course, recipe as described in [37]) by the neuropathologist (estimated fixation delay: ~45 min). After 24 h, the samples were cryoprotected with 30% sucrose (Sigma, S0389) in 1x DPBS −/− for approximately 48 h at 4 °C. Finally, samples were snap-frozen in Tissue-Tek O.C.T (Sakura, 4583) and stored at −80 °C until they were serially sectioned into 40 μm thick brain slices using a cryostat (Leica, CM3050 S). Free-floating slices were collected, washed 3x in 1x DPBS −/−, and then stored in cryoprotectant solution at −20 °C until further processing.

### Expansion Microscopy

#### Slice arrangement & anchoring

Mouse brain sections were obtained as described in the section on transcardial perfusion. After removal of the cryoprotectant solution, sectioned brain hemispheres of all animals (3x WT, 3x KO) were washed 3x in 1x DPBS −/− and arranged on top of the same super-frost plus microscope slide (VWR, 631-0108) to air-dry for approx. 10 min. This collective arrangement enabled the embedding of all samples to be compared within a single hydrogel as described below. The whole slide with the immobilized sections was first submerged in 10 μg/mL Acryloyl X-SE (ThermoFisher, A20770) in 1x DPBS −/− overnight at room temperature.

#### Gel polymerization

The gelation solution was adapted from [17] and contained 10.3% sodium acrylate (Sigma, 408220), 14.2% acrylamide (Sigma, A4058), 50 ppm N,N’-methylenebisacrylamide (Sigma, M1533) and 1x DPBS −/− once the polymerization was initiated by adding 15 ppm 4-Hydroxy-TEMPO (4HT; Sigma, 176141), 1.5 ppt TEMED (Bio-Rad, 1610801) and 1.5 ppt APS (ThermoFisher, 17874) to the pre-mixed monomer solution. The polymerization process was slowed by 4HT allowing for an extended incubation time of 30 min in ice-cold, but activated gelation solution in which the slide containing the brain slices was placed while shaking.

A droplet of 180 µL gelation solution was then placed into the well of a custom-built gelation chamber plate placed inside a fitted humid chamber (Fig. S15A, B). The microscope slide was mounted onto the droplet in the well, allowing potential air bubbles to escape by adding gelation solution through integrated channels, as needed. The mounted samples were then incubated within the humid chamber at 37 °C for 1 h to allow for full polymerization of the gelation solution, embedding the brain slices.

Afterwards, the gelation chamber was opened and excess gel surrounding the tissue slices was removed using a razor blade. At this point, an orthogonal view image was taken at a pre-defined height to document the pre-expansion dimensions of the gels used for calculating the expansion factor.

#### Denaturation & first expansion

The gels were then recovered into a solution of 50 mM Tris-BASE (Sigma, 93362), 200 mM NaCl (Sigma, 1.06404), 200 mM SDS (Carl Roth, 8029.3) in ddH2O (Millipore) at pH 9.0 (denaturation buffer) by starting to mobilize each of them with a brush dipped into the buffer and then slowly detaching them fully from the super-frost slide with a razor blade. All gels were then incubated in an Eppendorf tube filled with 1.0 mL of fresh denaturation buffer for 4 h at 80 °C in a Thermo-Block. Subsequently, the single hemispheres embedded within the hydrogel were transferred into big petri-dishes and washed 3×15 min, once overnight, and 1–2 additional times in ddH2O to remove any residual SDS and to complete a first round of full expansion. At this point, an orthogonal view image was taken at a pre-defined height to document the post-expansion dimensions of the gels used for calculating the expansion factor. Gels were washed 3-4x in 1x DPBS −/− to induce shrinkage. Afterwards the target brain regions were cut from each hemisphere, visualized by a bright LED-light placed right under a glass cutting surface. Dissection was simplified by the slight expansion of the gels when washed in 1x DPBS −/−.

#### Immunolabeling & final expansion

The regions of interest were first blocked in 3% BSA (Applichem, A1391) and 0.3% Triton X-100 (Sigma, T8787) in 1x DPBS −/− (blocking solution) for 3 h at room temperature on a shaker. Samples were then incubated with primary antibodies diluted in blocking solution for approx. 72 h at 4 °C on a shaker. Gels were washed 3 × 20–30 min in 1x DPBS −/− before incubation with secondary antibodies diluted in blocking solution, which was performed for approx. 24 h at 4 °C on a shaker. Again, gels were washed 3 × 20–30 min in 1x DPBS −/− following antibody incubation. The stained gels were then incubated with NHS ester tagged with BODIPY-FL (ThermoFisher, D2184) or Atto647N (Sigma, 18373) diluted to 20 µM in 1x DPBS −/− for 1 h at room temperature on a shaker. All immunolabeling conditions and antibody dilutions are documented in Table S2.

Finally, the samples were placed in a petri-dish, washed 3x in ddH2O and stored at 4 °C overnight, followed by 2 additional washes to complete the expansion of the hydrogels containing the regions of interest.

#### Imaging chamber

For imaging, the gels were mounted onto a #1.5H one-well chambered cover glass (Cellvis, C1-1.5H-N) previously coated with 0.1% PLL (Sigma, P2636) in ddH2O for 1 h at room temperature. To ensure the sample-side of the gel was facing the coated cover glass, samples had been checked for their orientation at a Thunder imaging system (Leica) equipped with a DFC9000 sCMOS camera, and a HC PL Fluotar 20x/0.4 air objective. The chambers containing the specimen were then filled with two-component silicone (Picodent, 13001000), first surrounding the edges of the sample, and then carefully filling up until the gel was completely covered. This process was completed within 4–5 min to avoid dehydration of the hydrogels. After the silicone had hardened after approx. 5-10 min, a wetted piece of paper tissue was placed on top, and the chamber was closed by fixing a standard microscope slide to its edges with super-glue. The construction of this custom imaging chamber (Fig. S15C–F) enabled drift- and dehydration-free prolonged imaging sessions at upright and inverted microscopes using a standard stage suitable for microscope slides.

#### Human samples

Human brain sections were obtained as described in the section on the fixation of human samples. Afterwards, the same procedures as described above were used to process human material.

#### Macroscopic expansion factor calculation

Images of pre- and post-expansion hydrogels were acquired as mentioned above. The ratio of pre- and post-expansion line measurements of the photographed gels was subsequently used as an indicator of the linear expansion factor achieved per embedded brain sample. For the monomer solution batch comparisons in Fig. S1 a two-sample Wilcoxon test with a significance level of alpha = 0.05 was used.

#### Confocal and STED microscopy

Confocal data was acquired using a laser-scanning confocal microscope (Leica TCS SPE II) equipped with a HC PL APO 63x/1.30 GLYC CORR CS2 (Leica) glycerol-immersion objective at a pixel size of 43 × 43 nm and z-steps of 120 nm.

**Fig. 1:**
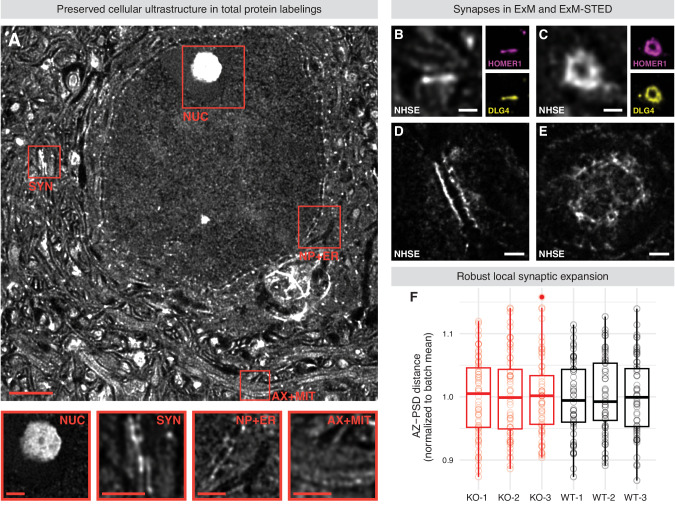
ExM and ExM-STED preserve cellular and synaptic ultrastructure. **A** Murine cortical tissue stained for total protein via NHS ester (NHSE) showcasing preserved cellular ultrastructure. Organelles are magnified and shown as inlets, corresponding to the boxes within the overview. Nucleoli (NUC) show intricate structuring and surroundings of varying protein density. The pre- and postsynaptic specializations including the synaptic cleft can be readily identified in the side view projection of a neuronal synapse (SYN). Nuclear pores spanning the nuclear envelope are visible in the direct neighborhood of the endoplasmatic reticulum (NP + ER). Axons traversing the imaged plane contain mitochondria (AX + MIT). Cortical synapses in side view (**B**) and en face (**C**) projection as imaged via confocal tenfold ExM. The main image shows the NHSE total protein staining, while inlets show immunolabelings targeting HOMER1 and DLG4 of the postsynaptic density. Cortical synapses in side view (**D**) and en face (**E**) projection as imaged via ExM-STED. As compared to **B** and **C** resolution is enhanced, showing the detailed ultrastructure of neuronal synapses. **F** Box plots showing the analysis of AZ-PSD distances to ensure comparable local synaptic expansion factors enabling further measurements. Color-coding is according to genotype (red for SHANK3-KO and black for WT mice). One-way ANOVA (*F* = 0.085, *p* = 0.995) with pairwise Tukey post-hoc analysis does not show significant differences (*p* > 0.99 in all pairwise comparisons; *n* = 60 per animal). Detailed statistical analysis, including the code to generate the plots is provided as R script in the supplementary materials. Scale bars represent 10 µm or 3 µm in the overview or inlets of **A**, respectively. Scale bars in **B**–**E** represent 1 µm.

STED microscopy was performed on a STEDYCON module (Abberior) fixed to an Axio Imager.Z2 (Zeiss) equipped with a Plan-NEOFLUAR 100x/1.30 (Zeiss) oil-immersion objective. Images were acquired in both confocal and STED mode. As dyes Abberior STAR ORANGE and Abberior STAR RED with an excitation laser of 561 nm (40% power) and 640 nm (20% power), respectively, were used in the genotype comparisons. A STED laser of 775 nm was used for both channels, set to 100% and 56.3% laser power, respectively. The pixel size was set to 25 x 25 nm with z-steps of 100 nm, a pixel dwell time of 10 µs and line accumulation of 5 in STED mode. Single synapses were acquired by specifying a rectangular region of interest. For visualizations in Fig. 1D and E, an NHS ester dye coupled to Atto 647 N was used. It was acquired using an excitation laser of 640 nm (10% power) and a STED laser of 775 nm (98.124% power). The pixel size was set to 25 x 25 nm with z-steps of 100 nm, a pixel dwell time of 10 µs and line accumulation of 5 in STED mode.

All images of human or murine cortical samples were acquired in layer 2/3, while murine striatal data was acquired in the neuropil of the caudate putamen. For cerebellar samples, images were acquired in the molecular layer. Imaging parameters in both settings were optimized to obtain signals with minimal crosstalk, while avoiding saturation. During tissue processing and microscopy, the experimenter was blinded for the genotypes of the animals.

### Image processing and analysis

Prior to any further processing all images acquired during confocal or STED microscopy were drift corrected and subsequently deconvolved using the respective implementations in the Huygens software (SVI).

#### Validation of comparable local synaptic expansion

To ensure comparable local synaptic expansion between animals, which were included in the same analysis, small pieces of hydrogel containing cortical tissue not needed for immunolabeling were separated and stained using an NHS ester dye coupled to Atto 647N to visualize cellular ultrastructure including the pre- and postsynaptic compartment. Images were then acquired in layer 2/3 after expansion using a confocal microscope. Using FIJI to process the previously deconvolved images, line selections were defined, spanning the side view synapses in a perpendicular manner, and passing both the AZ and PSD. A plot profile based on the pixel values along this line was derived and saved.

Using R for further analysis, a polynomial was fitted through the values derived from the plot profile and the respective peaks of the AZ and PSD were detected. Then the linear distance between the peaks on the x-axis was measured and defined as AZ-PSD distance of a synapse.

For the comparison of local expansion across different batches of monomer solution, the raw distances were compared using a two-sample Wilcoxon test at a significance level of alpha = 0.05.

For the analysis of local synaptic expansion across animals the raw distance values were normalized to the batch mean. The pooled normalized values of all batches were then used in a one-way ANOVA with Tukey post-hoc comparisons at a significance level of alpha = 0.05.

Additionally, for normalization steps in the subsequent analysis as described below, the AZ-PSD distances were averaged per animal and used as correction factor to account for potential differences in local expansion.

#### Feature-based synaptic shape analysis via confocal ExM

ExM in combination with confocal microscopy proved especially suitable for the analysis of general shape characteristics of synaptic protein assemblies, since resolution was sufficient for this purpose while also enabling the acquisition of thousands of synapses in a reasonable amount of time. Therefore, this approach was employed for the feature-based analysis of the synaptic DLG4 scaffold.

For initial thresholding a pixel classifier was trained on DLG4 immunolabelings using the FIJI-implementation of Labkit [38]. Representative images of 50 slices from 6 hyperstacks were concatenated into a single time series and labelled for training. The resulting classifier was applied to all stacks, included in the analysis. Low probability pixels of <0.2 were excluded. Subsequently, the thresholded probability maps were segmented and target values per synapse were derived using the 3D ROI Manager and 3D Suite Analysis [39]. Synapses touching the borders of XYZ were excluded. The measurements were then saved and further processed using R.

First the measurements were selected for analysis. Volume in pixels was converted to nm³ by multiplication with the acquired voxel size (43 × 43 x 120 nm) and normalized to the cubed mean AZ-PSD distance of the respective animal. Similarly, the smoothed mesh surface in pixels was converted to nm² by multiplication with the acquired pixel size (43 × 43 nm) and normalized to the squared mean AZ-PSD distance of the respective animal. The volume ratio was defined as the ratio of the summed volume of parts popping in or out of a fitted ellipsoid and the total volume of a synapse. Additionally, sphericity and five 3D Moments were included in the analysis. 3D Moments are shape descriptors invariant with respect to change of scale, translation and rotation, and describe the variations of the object from the ellipsoid [40, 41]. Finally, objects with volumes below the size of a cuboid defined by an edge-size of twice the theoretical resolution limit of a confocal microscope (2 × 200² x 500 nm) were rejected. The resulting value of 180.3 pixels was rounded to 200 pixels as exclusion threshold.

The resulting dataset of 9 variables was log-transformed, centered and scaled, and then processed by principal component analysis to reduce dimensionality to the first three principal components (PC1-PC3). On these principal components partional clustering via CLARA was performed. The optimal number of clusters was determined via Gap Statistics, which resulted in *k* = 6. Thus, each synaptic DLG4 scaffold detected was assigned to a cluster identity within the range of 1–6 according to its position in a space defined by PC1-PC3. The clusters were reordered to represent a running sequence within the observed distribution. To illustrate exemplary shapes in Fig. 3D the cluster medoids of the wild type sensory cortex were calculated. Following clustering, each synaptic scaffold was assigned a pseudotime value by fitting a principal curve through k1-6 in PC1-PC3. This value was interpreted as an indication of synaptic maturity. To define synaptic maturity according to morphological features, classical models of spine classification are often used, ranging from filopodia-like to “mature” mushroom-like spine morphology. These maturation steps generally result in a rising volume. Volume was also correlated to higher cluster identity and higher pseudotime values calculated in our analysis. However, volume alone only captures one dimension of the feature-set we derived from each postsynaptic shape analyzed in this study. Previous studies have shown that synaptic shape and PSD-morphology in particular predict AMPAR expression levels, where complex shapes showing segmentation and perforation correlate with high AMPAR levels and thus electrophysiological maturity [42]. Small PSDs within filopodia might even represent silent synapses completely lacking AMPAR expression [43]. Thus, pseudotime analysis was used as an unsupervised method, which considers all nine shape-based features to infer synaptic maturity from the observed continuum of synaptic shapes flowing from small spherical synapses to voluminous complex-shaped synapses. Notably, synaptic maturity can only be indirectly linked to pseudotime by which we infer it. For further analysis pseudotime was centered and scaled. A minimum of 1174 synapses was analyzed per animal in each condition.

For comparison of cluster composition across genotypes and brain regions a Chi-square test for independence (H0: Variables are independent/not associated, e.g., genotype does not influence synapse cluster identity) with post-hoc testing was performed. In both the analysis on genotypes and brain regions a post-hoc analysis to compare the deviation of single clusters from the expected proportions under H0 via standardized residuals and Bonferroni-corrected *p*-values was done. Pairwise Chi-square tests with Bonferroni-corrected p-values were performed for the direct comparison of brain regions (STR-SENS, STR-MOT, SENS-MOT). The X-squared-, df- and *p*-values are reported within the respective figures.

Additionally, statistical analysis on the original variables underlying the principal component analysis was performed. Since the assumption of normality was not fulfilled in all conditions, we applied a semi-parametric one-way MANOVA, which does not assume multivariate normality and covariance homogeneity. All variables and pseudotime were included in the testing, except for Moments 2–4, since they correlated strongly with Moment 1.

Following the MANOVA underlying multivariate genotype comparisons in each brain region a univariate Bonferroni-corrected post-hoc analysis to calculate p-values for all pairwise comparisons was conducted.

For the multivariate comparison of wild type brain regions (STR, SENS, MOT) firstly a semi-parametric MANOVA with pairwise multivariate post-hoc analysis, and secondly a univariate analysis with Bonferroni-corrected post-hoc analysis to identify variables that contributed to the rejection of H0 in the overall MANOVA, were performed. Finally, *p*-values reported in the respective figures were calculated via pairwise Benjamini-Hochberg-corrected post-hoc analysis of each univariate analysis.

The modified ANOVA-type statistic (MATS) and p-values representing the overall multivariate analysis are reported within the respective figure. p-value calculation was based on a parametric bootstrap resampling approach. To reduce the probability of type I errors in the presence of high sample sizes in this analysis the significance level was set to alpha = 0.01.

#### Analysis of subsynaptic particles via ExM-STED

Since the resolution was further increased by ExM in combination with STED microscopy, this approach proved especially suitable for the study of subsynaptic particles and their mosaic-like arrangements. However, the number of synapses, which can be imaged is both limited by time (each synapse is acquired separately) and availability of synapses that are oriented in parallel to the imaging plane (en face projections). This approach was thus used for the detailed analyis of subsynaptic particles.

Prior to analysis data acquired via ExM-STED were subject to further pre-processing. Before calculating the maximum intensity projection (MIP) of each stack (5–8 slices), a rolling ball background substraction with a ball size of 10 pixels was performed. Then a region of interest (ROI) surrounding each en face projected synapse was specified, which was used to create a mask selection. This selection served as input for the FIJI implementation of elastix [44–46] to transform and rotate channels 1 and 2 to a position of maximum correlation. This was done to ensure that slight deviations from orthogonality, when acquiring synapses en face, have minimal impact on correlation statistics calculated for both channels. Finally, the area outside of the specified ROIs was cleared prior to further analysis.

For thresholding a pixel classifier was trained on DLG4, HOMER1, and BSN immunolabelings using the FIJI-implementation of Labkit [38]. Representative MIPs were concatenated into a single time series per protein and labelled for training. The resulting classifier was applied to all stacks, included in the analysis. Low probability pixels of < 0.5 were excluded. Subsequently, the thresholded probability maps were saved as masks for further processing. Several types of measurements were calculated for the subsynaptic particles detected in the respective immunolabelings. A minimum of 16 synapses was acquired per animal in each condition. Since DLG4 was included in all conditions, synapses from the same brain region were pooled, roughly doubling the sample sizes in the respective analysis.

Firstly, the FIJI-plugin FindFoci [47] was used to detect single particles using the masks generated by thresholding of the probability maps as ROI. Basic properties such as size, intensity (total & average), and density (number of spots per area) were derived from this analysis. All values were averaged per synapse, normalized to the respective animal’s AZ-PSD distance averaged to the batch mean, and centered and scaled. The BIOP-implementation of the FIJI-plugin JACoP [48] was used to derive the Manders Coefficients as measure of colocalization, using the above mentioned masks as ROIs. M1 and M2 were measured per synapse. For statistical analysis of both basic properties and colocalization characteristics, we applied a semi-parametric one-way MANOVA. Following the MANOVA underlying multivariate genotype comparisons in each brain region a univariate Bonferroni-corrected post-hoc analysis to calculate p-values for all pairwise comparisons was conducted. The MATS and p-values representing the overall multivariate analysis are reported within the respective figure. p-value calculation was based on a parametric bootstrap resampling approach. The significance level was set to alpha = 0.05.

To further analyze spatial patterning of subsynaptic particles, the coordinates of each spot detected in the FindFoci workflow were processed using the spatstat package [49] in R. First a point pattern object was created from the given particle coordinates in a synapse. From these, the nearest neighbors (k1-20 for DLG4 and HOM1, k1-10 for BSN) and their distances from the respective reference point were calculated. All values were averaged per synapse and normalized to the respective animal’s AZ-PSD distance, which had been averaged to the batch mean. For statistical analysis, we applied a repeated-measures ANOVA with Benjamini-Hochberg-corrected post-hoc analysis to calculate p-values for all pairwise comparisons. The F-value and overall p-values are reported within the respective figure. The significance level was set to alpha = 0.05.

Additionally, we calculated the Ripley’s K-function for every point pattern object until a maximum radius of 20 pixels (500 nm, expanded units). The values were then averaged per radial distance, grouped by genotype, and visualized in Fig. S13. For statistical analysis, the summed deviation from complete spatial randomness per synapse was derived by subtracting the theoretical value from the K-estimate at each r, summing the resulting values and division by the number of measurements in each point pattern. Genotype comparisons of this measure were performed per immunolabeling and brain region via a two-sample Wilcoxon test at a significance level of alpha = 0.05.

To extend the analysis from measurements within single protein populations, we investigated the distances of spatially associated subsynaptic particles from co-stained channels. For this, a custom-written python program was used [50], which was based on the masks generated in the FindFoci workflow. Masks were processed pairwise. Every selection area in the first mask was checked for a corresponding area in the second mask. If such an overlap was found, both signals were considered as spatially colocalizing. Subsequently, the distances between the centre of masses of identified pairs was measured to infer the degree of association between postsynaptic and transsynaptic units. All values were averaged per synapse and normalized to the respective animal’s AZ-PSD distance, which had been averaged to the batch mean. Genotype comparisons of this measure were performed per condition (DLG4-HOM1 & DLG4-BSN in the STR & SENS) via a two-sample Wilcoxon test at a significance level of alpha = 0.05.

### Illustrations

Microscopy data in all figures has been subject to the Huygens-based deconvolution workflow. Additionally, MIPs or summed intensity projections (Fig. 1A and Fig. S4) of defined slices were calculated. Brightness and contrast were linearly adjusted for better visibility in all figures illustrating microscopic images. The inlets of Fig. 1A have been separately adjusted to best illustrate the organelles shown.

3D renderings of cluster medoids from the WT sensory cortex in Fig. 3D were prepared using Imaris Viewer (Oxford Instruments).

In the illustrated STED data, background was substracted using the rolling ball method with a ball size of 10 pixels. Figure 4A/E and Fig. 5 show STED data as analyzed, which includes transformation & rotation by elastix and clearing all pixels outside of a previously specified ROI around the outline of an en face projected synapse. Finally, plots and microscopic data were arranged in Adobe Illustrator.

### Statistical analysis

**Fig. 2:**
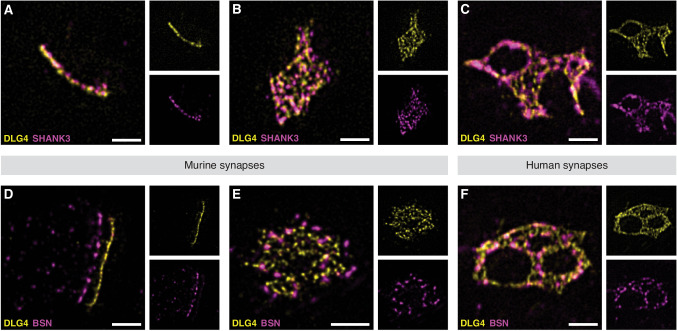
Localization of synaptic proteins visualized via ExM-STED. Staining for DLG4 and SHANK3 (**A-C**) or DLG4 and BSN (**D-F**) imaged via ExM-STED, showing a composite with merged channels in the main panel and inlets of the specific immunolabelings. **A** and **D** and **B** and **E** represent side view or en face projections of single murine synapses, respectively. **C** and **F** show en face projections of human synapses. DLG4 and SHANK3 are strongly associated, with SHANK3 being slightly more offset from the postsynaptic membrane (**A**). Together DLG4 and SHANK3 seem to be incorporated into a mosaic-like arrangement within the PSD, which can be appreciated in both en face projections of murine (**B**) and human (**C**) synapses. DLG4 and BSN are localized in the post- and presynaptic compartment separated by the synaptic cleft, respectively, with BSN showing a bell-like distribution within the presynaptic bouton (**D**). DLG4 and BSN are not as closely correlated in both en face projections of murine and human synapses (**E**, **F**), however seem to be organized in transsynaptic functional units possibly enabling effective neurotransmission. Scale bars represent 1 µm.

**Fig. 3:**
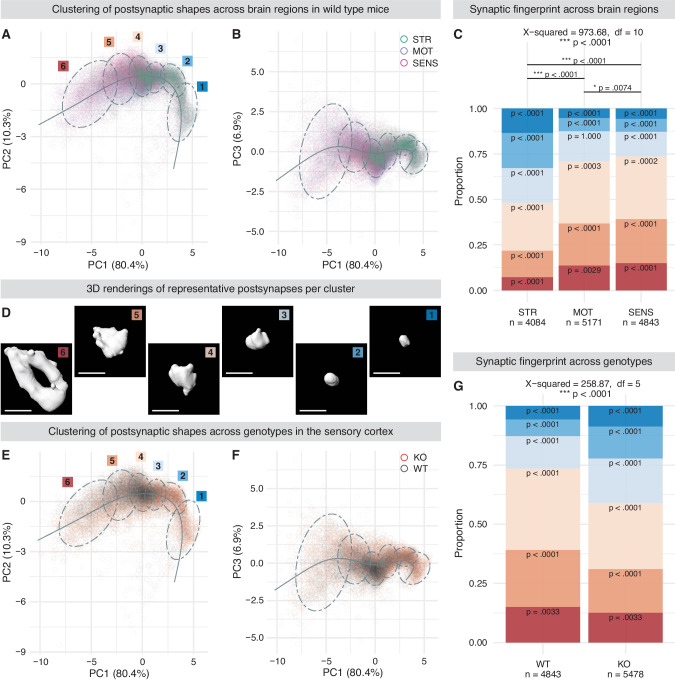
Morphological characteristics define brain region and genotype dependent synaptic fingerprints. **A/B**, **E/F** Scatterplots representing shapes of postsynaptic DLG4 scaffolds (one circle per shape) projected into a three-dimensional space defined by principal components (PC) 1-3. Shapes are categorized into six groups via partitional clustering as visualized by ellipsoids representing the 95% confidence level for a multivariate t-distribution. The fitted pseudotime curve is overlayed in each 2D scatterplot. Color-coding represents brain regions striatum (STR), motor (MOT) & sensory cortex (SENS) in (A/B) and genotypes wild type (WT) and SHANK3-KO (KO) in **E**/**F** as indicated in the respective legends. **D** Exemplary 3D renderings of the medoids from each cluster derived from the WT sensory cortex. Synaptic DLG4 scaffolds are spherical and small in cluster 1 and gain volume as well as complexity until cluster 6, where oftentimes perforations within the scaffold can be observed. Ellipsoids in **A**, **E** are labelled with the respective cluster identity shown in **D**. **C**, **G** Bar plots showing the proportional distribution of clusters as visualized and color-coded in **A**, **D** and **E**. **C** Chi-square test for independence shows that the investigated brain region does influence synapse cluster identity. Pairwise Bonferroni-corrected post-hoc comparisons reveal significant differences in cluster proportions among all brain regions analyzed and that all clusters deviate significantly from the expected proportions, except for cluster 3 in the motor cortex. The striatum presents with a clear overrepresentation of small/spherical clusters 1–3, while cortical regions are dominant in voluminous/complex shaped clusters 4-6, which is also visible in the scatterplots of **A**/**B**. **G** Chi-square test for independence reveals that genotype (WT vs. KO) influences synapse cluster identity in the sensory cortex and that all clusters deviate significantly from the expected proportions. SHANK3-KO mice present with an overrepresentation of small/spherical clusters 1-3, while voluminous/complex shaped clusters 4–6 are less abundant compared to WT animals, as visible in scatterplots (**E**/**F**). Sample sizes as number of synapses analyzed are indicated in (**C** & **G**). *p ≤ 0.01, ***p < 0.0001. Detailed statistical analysis, including the code to generate the plots is provided as R script in the supplementary materials. Scale bars in **D** represent 1 µm.

**Fig. 4:**
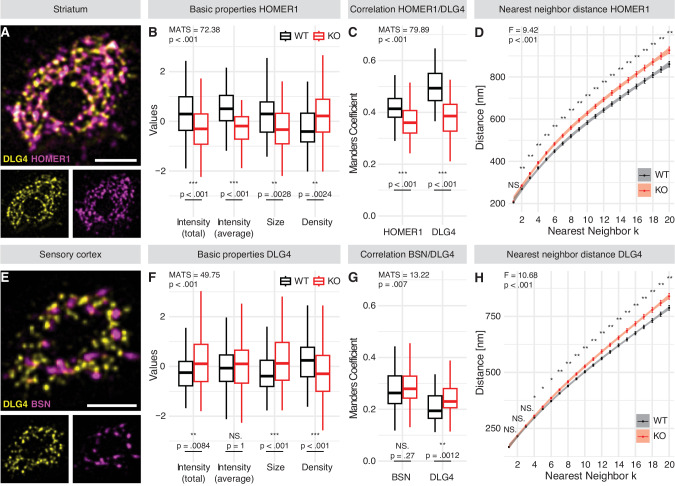
ExM-STED enables the detection of abnormal subsynaptic organization in SHANK3-KO mice. Representative en face images of single synapses immunolabeled for DLG4/HOMER1 (**A**) and DLG4/BSN (**E**), shown as analyzed. **A** was acquired in the striatal neuropil, while **E** was acquired in layer 2/3 of the sensory cortex of WT mice. **A** shows DLG4 and HOMER1 forming a postsynaptic web, while in **E**, DLG4 and BSN display less overlap. **B/C** Characterization of HOMER1 particles in the striatum of WT and KO mice, visualized by box plots. **B** shows that HOMER1 particles are smaller with lower protein amount, however, particle density per area is higher in KO animals compared to WT. Manders Coefficient-based correlation analysis in **C** reveals that both from the perspective of striatal HOMER1 and DLG4, colocalization is reduced under SHANK3-deficient conditions. (**F/G**) Characterization of DLG4 particles in the sensory cortex of WT and KO mice, visualized by box plots. **F** shows DLG4 particles are larger with higher total protein amount (however not when size-corrected), but lower particle density per area in KO animals compared to WT. Manders Coefficient-based correlation analysis in **G** reveals that from the perspective of cortical DLG4, colocalization with BSN is increased in SHANK3-KO. Nearest neighbor distance (NND) analysis of subsynaptic particles in the striatum (**D**) and sensory cortex (**H**) of WT and KO mice as visualized by point ranges representing the mean ± SEM per neighbor k and a fitted curve including its 95% confidence interval. Spatial pattern analysis via NND suggests a reduced association of striatal HOMER1-particles (**D**) or cortical DLG4 particles (**H**) to their respective neighborhood since distances to adjacent particles are increased. The values shown in (**B**, **F**) have been centered and scaled. In **B**/**C** and **F**/**G**, semi-parametric MANOVA with univariate Bonferroni-corrected post-hoc analysis was performed to calculate the p-values for all pairwise comparisons. Modified ANOVA-type statistics (MATS) and overall *p*-values are reported within the respective figure panels. Sample sizes as number of synapses analyzed in WT-/KO-mice are 54/59 in **B**, 54/59 in **C**, 129/139 in **F** and 69/73 in **G**. In **D** and **H**, repeated measures ANOVA with Benjamini-Hochberg-corrected post-hoc analysis was performed for all pairwise comparisons. The *F*-value and overall p-values are reported within the respective figure panels. Sample sizes as number of synapses analyzed in WT-/KO-mice are 54/59 in **D** and 128/138 in **H**. NS. *p* > 0.05, *p ≤ 0.05, **p < 0.01, ***p < 0.001. Detailed statistical analysis, including the code to generate the plots is provided as R script in the supplementary materials. Scale bars in (**A**, **E**) represent 1 µm.

**Fig. 5:**
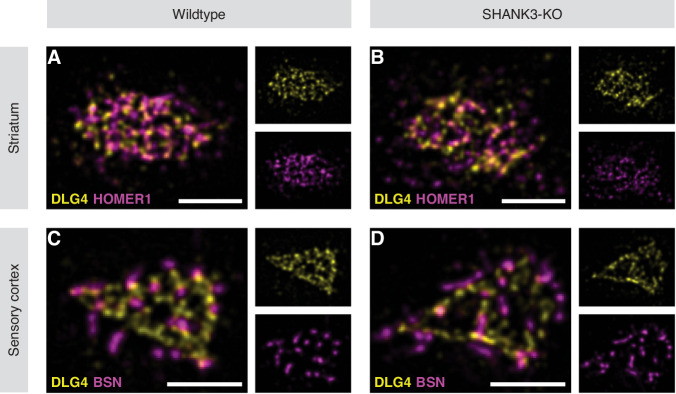
Visual comparison of subsynaptic organization across genotypes and brain regions. Representative en face images of single synapses immunolabeled for DLG4/HOMER1 (**A**/**B**) and DLG4/BSN (**C**/**D**) were acquired in the striatal neuropil and layer 2/3 of the sensory cortex, respectively. The genotype and brain region shown is indicated by row and column headings. **A**/**B** Reflecting the dominant subsynaptic changes evident from statistical analysis, representative images show that striatal HOMER1 particles are smaller with lower protein amount, but show higher density and an increased nearest neighbor distance under SHANK3-deficient conditions. Correlation of HOMER1- and DLG4-particles is also clearly reduced in SHANK3-KO animals. (**C**/**D**) Differences were not as pronounced when DLG4 and BSN were analyzed in the sensory cortex, however representative images already hint at the lower DLG4 particle density and higher nearest neighbor distance in KO animals as detected by statistical analysis. All images are shown as analyzed. Scale bars represent 1 µm in each panel.

The R programming language [51] was used to perform all statistical analyses and generate plots. Details are described in each figure legend and the analysis workflows above. The underlying datasets and R scripts for analysis and visualization are deposited at Edmond [52]. The folder structure is organized according to associated figures: Figure_NHSE-EF (Fig. 1/S1), Figure_SYNLOC (Fig. 2/S2, S3), Figure_REGSHAPE-SH3SHAPE (Fig. 3/S4–S8), Figure_SH3STED (Figs. 4–5/S9–S14), Figure_S15. In each folder all images, which were used in the figures are also included.

### Software and programming languages

R 4.2.0 [51] with the additional packages tidyr 1.2.1 [53], plyr 1.8.8 [54], dplyr 1.0.10 [55], reshape2 1.4.4 [56], purrr 0.3.5 [57], parameters 0.20.2 [58], cluster 2.1.4 [59], pracma 2.4.2 [60], slingshot 2.4.0 [61], spatstat 3.0-3 [49], chisq.posthoc.test 0.1.2 [62], MANOVA.RM 0.5.3 [63], mvnormtest 0.1-9 [64], onewaytest 2.6 [65], rstatix 0.7.1 [66], factoextra 1.0.7 [67], ggplot2 3.4.0 [68], wesanderson 0.3.6 [69], and ggsignif 0.6.4 [70].

In the measurements of pairwise distances of overlapping subsynaptic particles Python 3.5.1 (Anaconda distribution) and imaging features of the scikit-image module were used for loading and saving of image files [71].

Additional software used for image processing and preparation of illustrations included Huygens 22.04, FIJI (ImageJ 2.3.0/1.53t confocal & 2.9.0/1.53t STED), Labkit [38], Elastix [44–46], JACoP [48], FindFoci [47], Imaris Viewer (10.0.0), and Adobe Illustrator (2021 25.0.0).

## Results

### Optimized protocol of Expansion Microscopy preserves general cellular and synaptic nanostructure and enables detailed analysis

#### Expansion factor validation and preservation of cellular ultrastructure

To enable nanostructural analysis of synapses in big volumes at high speed and precision, we combined a validated gel recipe for single-step ten-fold expansion [17] with tissue processing by cryostat cutting allowing for the use of long-term stored materials, prolonged denaturation, and post-denaturation antibody labeling using an optimized staining regimen. Additionally, we designed a novel gelation chamber plate to minimize inter-experiment variation, and a custom gel mounting strategy, which prevented gel drift and dehydration during imaging. These adaptions and advantages intrinsic to ExM (e.g., low optical aberration, reduced autofluorescence) resulted in high signal-to-noise ratios suitable for STED microscopy. Additional pre-processing via deconvolution and drift-correction facilitated automatized segmentation of synaptic markers with subsequent multidimensional shape-based clustering for large-scale data analysis.

Our optimized protocol successfully preserved cellular ultrastructure in murine brain slices, as evidenced by the NHS-ester based visualization of various cellular organelles, including synapses and their geometrical complexity (Fig. 1A). Total protein labeling via NHS-ester chemistry also provided ultrastructural context to specific antibody-based staining, as shown in side view and en face projections of the postsynaptic markers HOMER1 and DLG4 (Fig. 1B, C). Moreover, the combination of ExM and STED microscopy further improved the resolution of synapses (Fig. 1D, E). If an expansion factor of tenfold and an approximate lateral resolution of 30-40 nm in STED microscopy is assumed, then the effective resolution of ExM-STED is approximately 3–4 nm, which is further improved by deconvolution.

To ensure comparable local synaptic expansion for each animal used in this study, peak-to-peak distances between the postsynaptic density (PSD) and active zone (AZ) of synapses were measured. This distance did not differ significantly between animals, which were directly compared in an analysis, confirming that our protocol is robust and suitable for comparisons without being influenced by unequal sample expansion (Fig. 1F). However, expansion measured at both macroscopic and synaptic distance levels varied slightly across different batches of monomer solution (Fig. S1A, B). One batch was used in confocal ExM experiments, the other in ExM-STED experiments. In Table S1, an overview illustrating, which monomer solution batch was associated to each figure or analysis, is provided.

Since AZ-proteins have been reported to assemble at a distance of 30-60 nm to the PSD, with proteins associated to dense projections (DPs) like RIM1 clustering at a distance of about 50 nm [72–74], the AZ-PSD distances measured in this study (Batch A: 536.7 ± 2.4 nm; Batch B: 510.4 ± 2.4 nm) suggest a local expansion factor of approximately tenfold. However, from our point of view any measurement, which might be indicative of the expansion factor lacks the necessary precision to allow confident conversion to “pre-expansion” units. Thus, all distances, areas, and volumes showcased or analyzed were not corrected for expansion.

#### Localization of synaptic proteins using ExM and ExM-STED

Next, ExM-STED was applied to visualize various synaptic targets in brain slices, revealing the intricate organization of SSDs. SSDs appeared to be composed of multiple particles serving as building blocks of trans-synaptic nanocolumns both visible in en face and side view projections (Fig. 2). These proteins were observed to form mosaic-like arrangements in en face projections (Fig. 2B, C, E, F), which were not resolved in confocal ExM (Fig. S2). Confocal ExM showed the PSD in great detail, oftentimes including perforations and complex shapes in bigger, more mature synapses (Fig. S2). ExM-STED showed that below this general distribution within the postsynapse, DLG4 as membrane-associated postsynaptic scaffolding protein organizes in regularly arranged subsynaptic particles, which differentially associate with other proteins within the synaptic mosaic like SHANK3, HOMER1 and BSN (Fig. 2; Fig. S3A, D). These subsynaptic particles were observed in both murine brain slices (Fig. 2B, E; Fig. S3A, D) and human archival cortex samples (Fig. 2C, F). The latter were derived from the resection of an epileptic seizure focus. ExM-STED additionally revealed a close association of both SHANK3 and HOMER1 with DLG4 in a web-like arrangement within the murine PSD, suggesting their orchestrated participation in its formation and maintenance (Fig. 2A, B; Fig. S3A, D).

Subsynaptic particles were also present in the AZ as visualized via BSN (Fig. 2D–F). These were distributed at larger distances than their postsynaptic counterparts and showed repulsion to colocalization with DLG4 particles when viewed en face (Fig. 2E). This might correlate with vesicle fusion sites’ positioning between DPs and directly opposing postsynaptic sites of receptor clustering [20]. Side view acquisition confirmed the pre- and postsynaptic localization of BSN and DLG4, respectively (Fig. 2D). CAMK2A and GRIA2 showed similar subsynaptic patterns (Fig. S3B, E), with CAMK2A also displaying clear pre- and postsynaptic pools, supporting recent findings suggesting a role for CAMK2 at the presynapse [75, 76]. In the cerebellum, comparable structures were visualized (Fig. S3C, F) using a combination of postsynaptic HOMER3, and VGLUT1 as marker of presynaptic vesicles.

From this initial validation we concluded that confocal ExM in combination with ExM-STED not only preserves cellular ultrastructure, but also offers a powerful tool to elucidate the complex nanostructural properties of synapses and their potential role in brain function and disease. Additionally, as accessible, scalable, and cost-effective imaging technique ExM and ExM-STED could provide novel insights into cellular organization and function in other areas of neuroscience and beyond.

### Nanostructural analysis of synaptic shapes and SSDs enables detection of anomalies in models of neuropsychiatric disease

#### Feature-based cluster analysis of synapses across brain regions and genotypes

To further evaluate the capability of ExM-derived datasets to capture biologically relevant information, we first compared the shape of postsynaptic DLG4 scaffolds localized either within the striatum, motor, or sensory cortex of WT mice (Fig. 3A–C). DLG4 scaffolds were categorized via partitional clustering using principal components derived from a feature set describing their three-dimensional properties. Six clusters were identified, with more voluminous and complex synapse shapes as the cluster identity progressed from 1 to 6. Exemplary shapes of cortical DLG4 scaffolds are illustrated in Fig. 3D, which have been selected by calculation of the respective cluster medoids (images underlying the 3D reconstruction are shown in Fig. S4). As additional measure, pseudotime analysis was employed to assign each shape to a so-called timepoint within the observed distribution, with rising pseudotime indicating increased synaptic maturity (Fig. S5).

Brain regions were found to have a distinct “synaptic fingerprint” based on their DLG4 scaffold shapes. Clusters containing larger and more complex shapes were overrepresented in layer 2/3 of the motor and sensory cortex when compared to the striatal neuropil, which was dominated by smaller, spherical, and less mature scaffold shapes (Fig. 3A–C; Fig. S6A).

Since ExM confirmed our initial hypothesis that it could describe biologically relevant differences by the identification of shape-based cortical and subcortical synaptic fingerprints we next tested whether genotype-dependent effects might be detectable in a murine model of syndromic neurodevelopmental disorder. We thus compared postsynaptic DLG4 scaffolds of WT and ex11 | SH3 SHANK3-KO mice (three animals per genotype; a minimum of 1174 synapses were analyzed per animal). Indeed, differences between genotypes could be identified according to this synaptic profiling in all brain regions analyzed (Fig. 3E–G; Fig. S7 & S8). SHANK3-deficiency generally led to an overrepresentation of postsynapses in small/spherical clusters 1-3 and an underrepresentation in larger/complex-shaped clusters 4-6. The knockout of SHANK3 thus generally led to a phenotype, which approached a distribution similar to that observed in the WT-striatum. This effect was pronounced in the cortical regions, which had presented with more complex synapses under baseline conditions (Fig. 3A–C). Comparison of genotypes via MANOVA and subsequent univariate posthoc-analysis using the raw shape features underlying the PCA-based clustering confirmed these results in all brain regions, also showing a decreased pseudotime value of SHANK3-deficient postsynapses, indicating lower synaptic maturity along the observed distribution (Fig. S6B–D).

#### Detailed profiling of SSDs reveals genotype-dependent alterations

Since the overall shape of postsynapses seemed to be affected by SHANK3-KO (Fig. 3E–G; Fig. S7 & S8), we next asked whether ExM-STED might be suitable to detect more intricate alterations of synaptic architecture as visualized in en face projections (Fig. 4A, E; three animals per genotype; a minimum of 16 synapses were analyzed per animal). In fact, already basic properties of the synaptic nanoscale mosaic were altered. For instance, in the striatum, but not sensory cortex of SHANK3-deficient mice, subsynaptic HOMER1 particles were smaller, with lower protein amount as measured via intensity. However, a higher density of particles per area was observed, hinting at an increased postsynaptic fragmentation upon SHANK3-deficiency (Fig. 4B; Fig. S9A). Conversely, DLG4 and BSN as proteins not directly interacting with SHANK3, generally presented with inverted trends. Subsynaptic DLG4 particles in the sensory cortex were bigger, showed increased total but not average protein amount and a decreased density pointing towards increased protein clustering (Fig. 4F). These changes were less pronounced in the striatum (Fig. S9B). Similar trends were observed when subsynaptic BSN particles were analyzed in the striatum or sensory cortex (increased intensity and size, decreased density; Fig. S9C, D). Thus, both pre- and postsynaptic compartments are affected, with different synaptic protein populations exhibiting unique responses to SHANK3-deficiency, which might reflect both their general functionality at the synapse and relationship to SHANK3 itself. Supporting this notion, striatal HOMER1 as direct interaction partner of SHANK3 [28] known to form a polymeric network with SHANK-proteins [77], showed the most prominent alterations (Fig. 4B). Notably, regional differences were also identified in the ExM-STED based analysis. While subsynaptic BSN particles presented with equal properties across brain regions (Fig. S10A), both DLG4 and HOMER1 particles were smaller but more numerous in the sensory cortex when compared to the striatum of WT mice (Fig. S10B, C).

In addition to these baseline alterations and consistent with the dominant changes of striatal HOMER1, colocalization of HOMER1 and DLG4 was strongly reduced in this brain region under SHANK3-deficient conditions, but not in the sensory cortex (Fig. 4C; Fig. S11A). Conversely, colocalization of subsynaptic DLG4 particles with presynaptic BSN was increased in both the sensory cortex and striatum (Fig. 4G; Fig. S11B). These changes in spatial correlation suggest that proteins within the PSD, as well as functional units spanning the PSD and AZ might be misaligned, potentially hindering effective neurotransmission.

To investigate possible alterations of spatial molecular patterns a nearest neighbor distance (NND) analysis was performed, which measures the distance of each given object to its nearest neighbor k. Subsynaptic particles revealed a closer association to neighborhood objects in WT, when compared to SHANK3-KO animals for both HOMER1 (striatum and sensory cortex; Fig. 4D; Fig. S12A) and DLG4 (sensory cortex, but not striatum; Fig. 4H; Fig. S12B). No changes were observed in both brain regions when BSN was analyzed (Fig. S12C, D). Point pattern analysis via K-estimate curves additionally revealed unique spatial pattern characteristics for each of HOMER1, DLG4 and BSN (Fig. S13A–F). When distances between overlapping particles of co-stained proteins were analyzed under SHANK3-deficient conditions, we found a significant increase for both HOMER1-DLG4 or BSN-DLG4 particle pairs in the striatum and sensory cortex (Fig. S14A–D). This further hints at a functional decoupling of associated protein clusters within a trans-synaptic nanocolumn, which are crucial for effective neurotransmission. Representative images of striatal and cortical synapses of both genotypes acquired via ExM-STED are shown in Fig. 5.

## Discussion

In this study, we demonstrate that a single-step ten-fold ExM protocol enables high-quality imaging and automated analysis of synapses at nanoscale-resolution using both confocal and STED microscopy. This approach enabled detection of previously unknown fingerprints of postsynaptic shapes specific to single brain regions, and the visualization and analysis of novel building blocks of subsynaptic organization. By applying the resulting experimental and computational workflow to the ex11|SH3 model of SHANK3-deficiency, which mimics ASD and PMDS, we additionally provide evidence for alterations in synaptic shape and subsynaptic particle organization in SSDs that may underlie the abnormal neurotransmission observed in these animals [35].

ExM offers several innate advantages over traditional imaging methods, such as improved signal-to-noise ratios, practically aberration-free imaging due to the transparency of the gel, reduced autofluorescence, and compatibility with conventional fluorescence microscopy systems [78, 79]. While the original ExM protocol had enabled lateral resolutions of 70 nm [12], advancements of the technique achieved 20-25 nm resolution through iterative expansion or increasing the expansion factor [15, 17, 80–82]. Recently, the combination of ExM with other SRM-techniques further pushed resolution below 10 nm [83, 84], including imaging of synapses [85, 86]. In this context, our specific approach combined a previously validated gel recipe for ten-fold ExM [17] with several adaptations to the protocol, including a custom gelation chamber plate and gel mounting strategy to allow for a highly standardized experimental workflow. The mounted gels are both compatible with inverted confocal microscopes and an upright STEDYCON system. The combination of ExM with STED and deconvolution employed in this study further enhances resolution to single-digit nanometers, allowing for unprecedented insights into the intricate organization of synaptic nanostructure in both murine and human brain tissue. Despite these benefits, potential limitations of ExM and ExM-STED include uncertainty regarding the exact expansion factor achieved during sample processing and the possibility of artifacts introduced by the expansion process. In our study, we addressed this by measuring peak-to-peak distances between PSDs and AZs, ensuring that the samples were comparable in terms of local expansion. Future improvements could involve cryofixation to minimize potential distortions or artifacts [87, 88] and further enhance resolution via combination with other SRM techniques, like advanced 3D-STED or MINSTED systems [89, 90].

The application of ExM in this study revealed genotype-dependent alterations in postsynaptic DLG4 scaffolds characterized by a shift towards lower volume and more spherical shapes in the ex11|SH3 model of SHANK3-deficiency. We also found that shape profiles or synaptic fingerprints were specific to certain brain regions, which might be influenced by the fact that the SHANK3-interactome varies between them [91]. Another factor associated to the *Shank* gene family possibly influencing synaptic shaping could be their specific expression patterns, where *Shank3* clearly dominates in the striatum [92].

ExM-STED further unveiled synaptic protein assemblies as mosaic-like arrangements in both human and murine brain tissue, which were not described previously. These are likely to represent molecular correlates of interwoven filaments of protein scaffolds surrounding receptor complexes in the PSD as observable via electron microscopy [93–95]. This technique’s molecular specificity in characterizing subsynaptic particles showed profound effects of SHANK3-deficiency on the organization of pre- and postsynaptic mosaics. Striatal HOMER1, a direct interaction partner of SHANK3, revealed the most prominent alterations also matching previous findings [35, 96]. Since the HOMER-binding motif is not directly affected by deletion of exon 11, it is likely that global destabilization and downregulation of SHANK3 led to the observed changes. The fact that anomalies related to subsynaptic HOMER1 particles were only present in the striatum and not the sensory cortex might also stem from regionally defined SHANK3-interactomes [91] or the capability of the cortex to compensate for SHANK3-loss by upregulation of SHANK1 and SHANK2, which are expressed at lower levels in the striatum [92]. DLG4 and BSN generally presented with inverted trends, which were less pronounced in the striatum but evident in the sensory cortex. Notably, we identified a misalignment of functional units within the PSD, but also spanning the PSD and AZ, which might hinder effective neurotransmission. DLG4 anchors receptors to the postsynaptic membrane, while vesicle fusion has been hypothesized to take place between DPs of the AZ [20, 97, 98]. Consequently, BSN should not align perfectly with DLG4, allowing vesicle fusion sites to directly oppose receptor anchoring sites provided by the DLG4 scaffold. Increased general colocalization of DLG4 and BSN as observed under SHANK3-deficient conditions could thus affect the kinetics of physiological neurotransmission. The observed dissociation of HOMER1 and DLG4 within the PSD might further contribute to synaptic dysfunction. These changes thus provide evidence for PSD-fragmentation, as well as misalignment and functional decoupling of the pre- and postsynaptic compartment in SHANK3-KO mice.

We present results that expand on the concept of SSDs and trans-synaptic nanocolumns as building blocks of synapses [20, 99, 100] and provide a comprehensive characterization of nanostructural abnormalities in synaptic shapes and SSDs across multiple brain regions in SHANK3-KO mice. Given previous evidence on the influence of synaptic shape [42, 43, 101–107] and SSDs [18–22, 24] on receptor distribution, and synaptic transmission and plasticity, the observed changes likely represent morphological correlates of previously described electrophysiological abnormalities in SHANK3-deficient animals. Most evidence across the variety of murine models points towards an impaired postsynaptic plasticity [35, 108–115]. Deficient mechanisms of plasticity in the striatum, which are linked to a disrupted interaction of mGluR5 and HOMER, were also found in the exonic deletion model (ex11|SH3) used in this study [35]. Notably, altered mGluR5-HOMER dynamics and the resulting impact on synaptic plasticity were also described in other models of SHANK3-deficiency [116, 117], possibly affecting the influence of perisynaptic mGluR5 on synaptic function [118]. An altered organization of the subsynaptic HOMER1-mosaic might thus represent the structural underpinning of this deficient interaction with glutamate receptors and contribute to the disruption of synaptic plasticity. This study thus adds evidence to the notion that synaptic dysfunction plays a pivotal role in the pathophysiology of SHANK-associated neuropsychiatric disorders such as ASD and PMDS, which might contribute to the variety of cognitive and behavioral phenotypes observed in SHANK3-KO mice [119]. Our findings also highlight the importance of considering not only global synaptic organization, but also the intricate substructure underlying SSDs to characterize their composition under physiological and pathological conditions such as SHANK3-related syndromes, which was enabled by ExM-STED.

Overall, our study establishes a strong foundation for future research aimed at deciphering the role of synaptic properties in neuropsychiatric disorders and highlights the potential of ExM-STED as a powerful tool for studying synaptic nanostructure. These insights also have potential implications for the evaluation of therapeutic strategies targeting synaptic aberrations since the nanostructural alterations described may serve as readouts for future studies.

## Supplementary information

Supplementary Material

## Data Availability

All data are available in the manuscript and deposited at Edmond [52].
