# Supplementary material for: Combined expansion and STED microscopy reveals altered fingerprints of postsynaptic nanostructure across brain regions in ASD-related SHANK3-deficiency
Source: Mol Psychiatry. 2024 Apr 22;29(10):2997–3009. doi: 10.1038/s41380-024-02559-9 (PMC11449788; doi:10.1038/s41380-024-02559-9)
Supplement: Supplementary file 1 — Supplementary Material [file 41380_2024_2559_MOESM1_ESM.pdf]

Supplementary Information for

Combined expansion and STED microscopy reveals altered fingerprints of postsynaptic nanostructure across brain regions in ASD-related SHANK3-deficiency

Jan Philipp Delling*, Helen Friedericke Bauer, Susanne Gerlach-Arbeiter, Michael Schön, Christian Jacob, Jan Wagner, Maria Teresa Pedro, Bernd Knöll, Tobias M. Boeckers*

*Corresponding author: tobias.boeckers@uni-ulm.de, jan.delling@alumni.uni-ulm.de

The PDF file includes:

Figures S1 to S15

Table S1 and S2

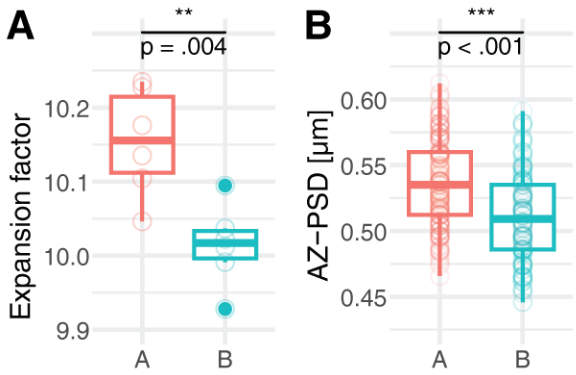

Fig. S1. Expansion factor varies between batches of monomer solution.

(**A**) Box plots showing the linear expansion factor as measured macroscopically via images of the gels before and after expansion. Color-coding is according to monomer solution batches A & B, each circle representing one animal used in this study (n = 6 per batch). (**B**) Box plots showing the analysis of AZ-PSD distances to compare local synaptic expansion factors between batches of monomer solution. Color-coding is according to monomer solution batches A & B, each circle representing one synaptic distance measurement taken from a total of 6 animals (n = 30 per animal and batch). Two-sample Wilcoxon tests show significant differences between batches A & B in both macroscopic (A) and local (B) analysis of the expansion factor, being slightly higher in batch A (n = 6 per batch). ** $p < .01$, *** $p < .001$. Detailed statistical analysis, including the code to generate the plots is provided as R script in the supplementary materials.

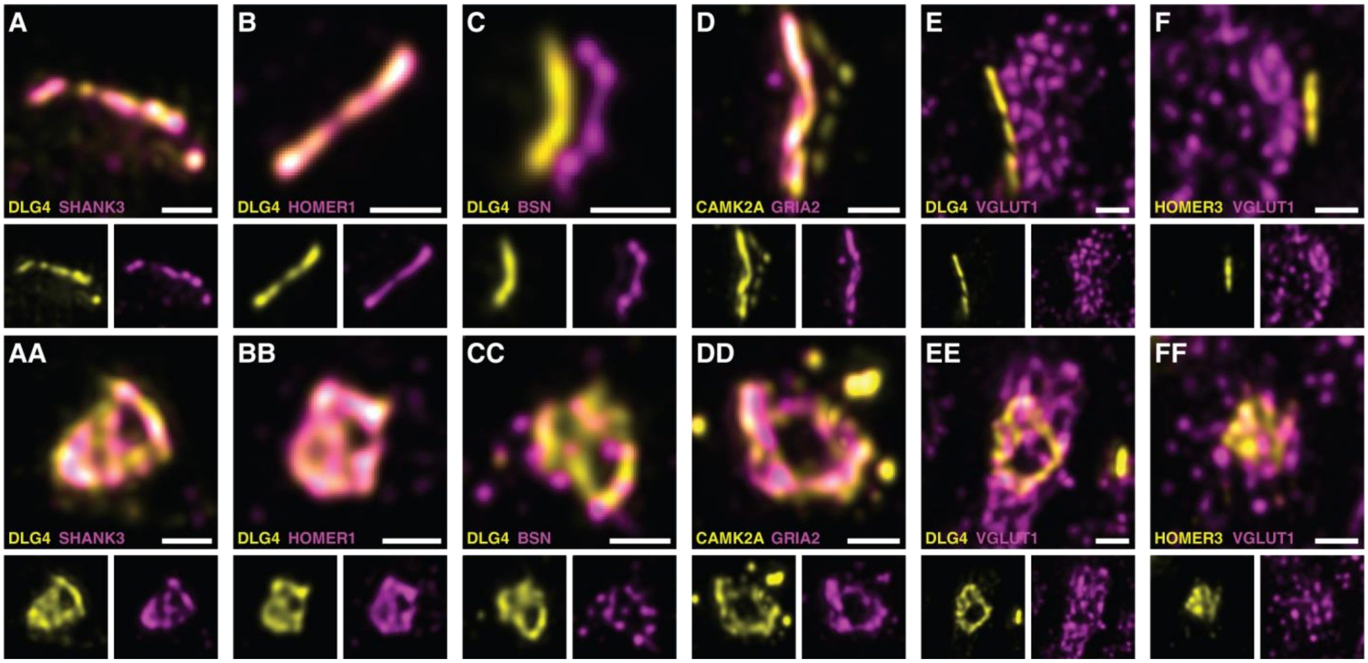

Fig. S2. Localization of synaptic proteins visualized via confocal ExM.

Staining for DLG4/SHANK3 (**A/AA**), DLG4/HOMER1 (**B/BB**), DLG4/BSN (**C/CC**), CAMK2A/GRIA2 (**D/DD**), DLG4/VGLUT1 (**E/EE**) and HOMER3/VGLUT1 (**F/FF**) imaged via confocal ExM, showing a composite with merged channels in the main panel and inlets of the single immunolabelings. (A-F) and (AA-FF) represent side view or en face projections of murine synapses, respectively. DLG4 is highly colocalizing with SHANK3 (A/AA) and HOMER1 (B/BB) within the postsynaptic density (PSD) of cortical synapses localized in layer 2/3. DLG4 and BSN (C/CC) or VGLUT1 (E/EE) within cortical synapses of layer 2/3, and HOMER3 and VGLUT1 (F/FF) in the cerebellar molecular layer are clearly localized in the post- and presynaptic compartment separated by the synaptic cleft, respectively. CAMK2A shows both pre- and postsynaptic protein pools, while GRIA2 is localized at the postsynaptic side of cortical synapses (D/DD). En face projections of synapses (AA-FF) often show perforations within the structure of the PSD. Scale bars represent 1 µm.

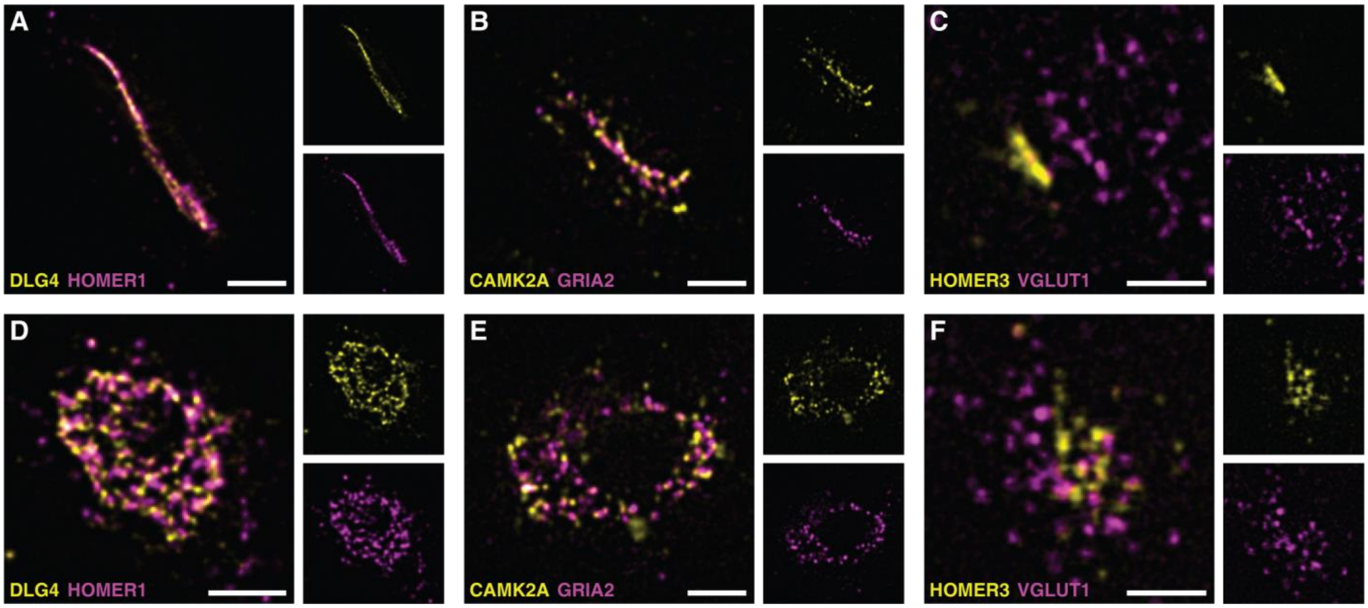

Fig. S3. Localization of synaptic proteins visualized via ExM-STED.

Staining for DLG4/HOMER1 (**A & D**), CAMK2A/GRIA2 (**B & E**), and HOMER3/VGLUT1 (**C & F**) imaged via ExM-STED, showing a composite with merged channels in the main panel and inlets of the single immunolabelings. (A-C) and (D-F) represent side view or en face projections of murine synapses, respectively. DLG4 and HOMER1 (A/D) are strongly associated, being incorporated into a mosaic-like arrangement within the postsynaptic density of cortical synapses within layer 2/3. CAMK2A shows both pre- and postsynaptic protein pools, while GRIA2 is localized at the postsynaptic side of cortical synapses within layer 2/3 (B/E). The en face projections reveal an association between subsynaptic particles, similar to DLG4 or HOMER1 (E). Cerebellar (molecular layer) HOMER3 and VGLUT1 are localized in the post- and presynaptic compartment separated by the synaptic cleft, respectively, with both protein populations presenting with an intricately organized subsynaptic structure (C/F). Scale bars represent 1 µm.

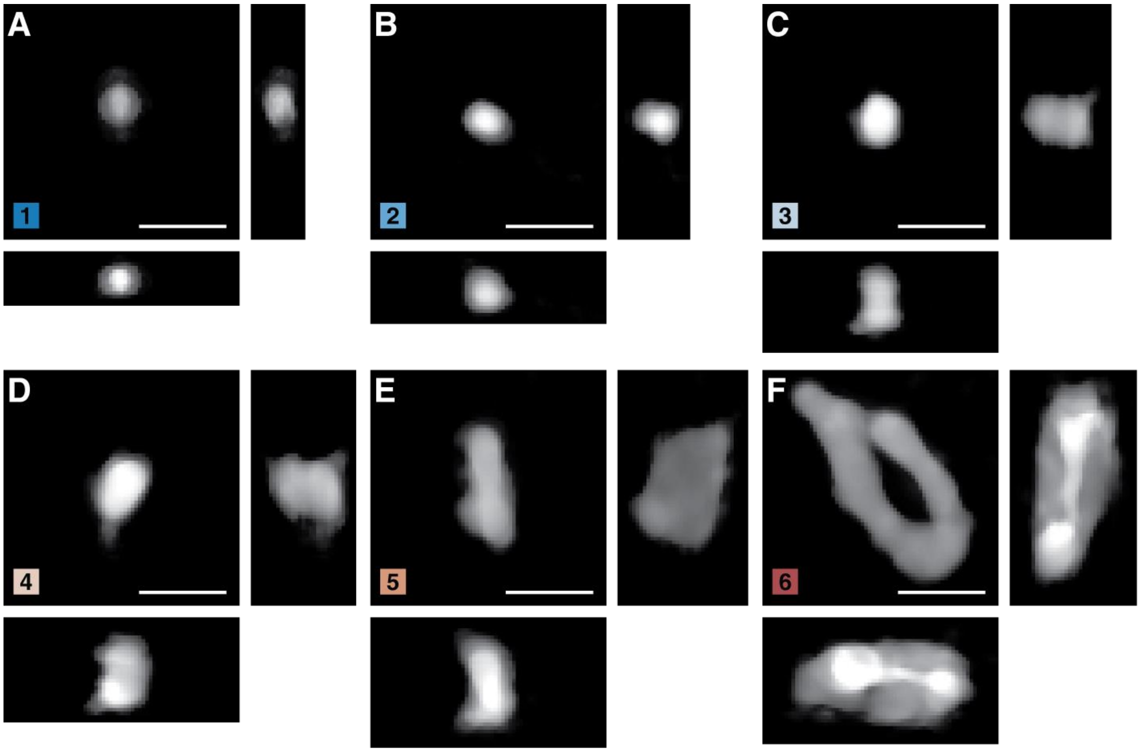

Fig. S4. Representative images of DLG4-stained postsynapses across identified clusters.

(**A-F**) Summed intensity projections of the images underlying the 3D renderings of representative postsynapses from each cluster 1-6 derived from the wildtype sensory cortex shown in Fig. 3D. Main images are supplemented by XZ- and YZ-views at the bottom and right-hand side, respectively. Each panel is labelled with the corresponding cluster identity. All scale bars represent 1 µm.

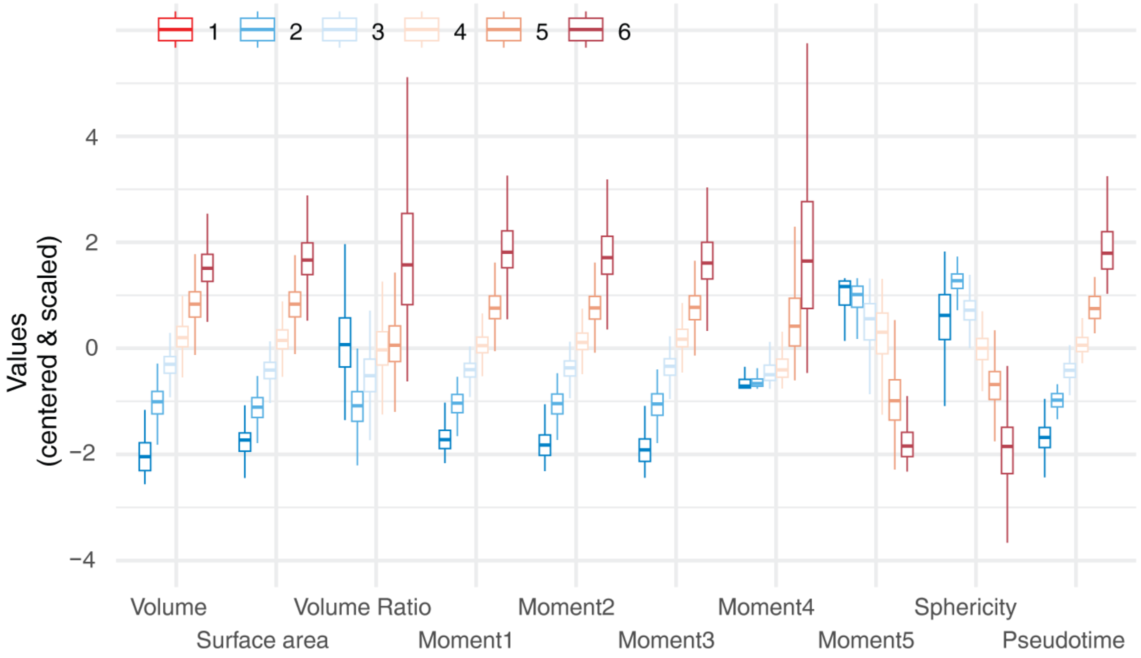

Fig. S5. Univariate synaptic profiles across cluster identity.

Box plots illustrating the profiles of postsynaptic DLG4 scaffold shapes across cluster identity. Each variable underlying the principal component analysis, and pseudotime values are shown. A positive correlation with rising cluster identity (1-6) is visible for Volume, Surface area, Moment1-4 and Pseudotime. A negative correlation is present for the values of Moment5. Volume ratio peaks initially at cluster 1 before increasing linearly to its maximum at cluster 6. Clusters 4/5 reach levels similar to that of cluster 1. Sphericity shows an inverse distribution when compared to Volume Ratio, peaking at cluster 2, then decreasing across clusters 3-6. Both Volume Ratio and Sphericity indicate that synaptic scaffolds of cluster 1 exhibit more irregular shapes than clusters 2 & 3, despite the lowest Volume being in cluster 1. The values shown have been centered and scaled. No statistical tests were conducted. Detailed information, including the code to generate the plots is provided as R script in the supplements.

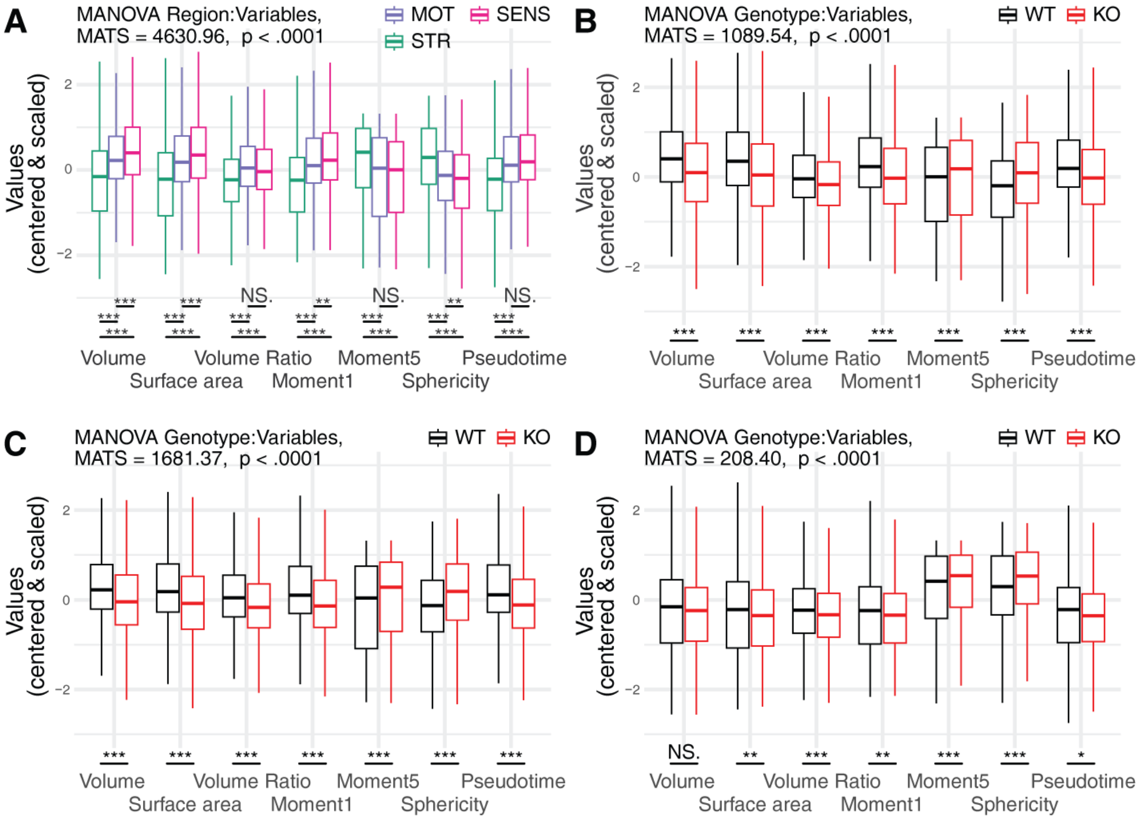

Fig. S6. Univariate synaptic profiles across brain regions and genotypes.

Box plots illustrating the profiles of postsynaptic DLG4 scaffold shapes across brain regions (**A**) and genotypes (**B-D**). A selection of variables underlying the principal component analysis, and pseudotime values are shown. (A) Multivariate analysis indicates a significant influence of the brain region on the analyzed outcome variables overall and in all pairwise comparisons. All variables contributed to the rejection of H0. Pairwise univariate comparisons show that the sensory (SENS) and motor cortex (MOT) differ significantly in Volume, Surface area, Moment 1 and Sphericity. SENS exhibits higher volume, surface area and value of Moment 1, but lower sphericity than MOT. The striatum (STR) significantly differs from cortical regions in all variables analyzed, presenting lower values in Volume, Surface area, Volume Ratio, Moment 1 and Pseudotime, in addition to higher values in Moment 5, and Sphericity. (B/C) Multivariate analysis reveals a significant influence of genotype on the analyzed outcome variables in the SENS (B) and

MOT (C). Post-hoc analysis on univariate comparisons shows that all variables contribute to the rejection of H0. SHANK3-KO leads to lower values in Volume, Surface Area, Volume Ratio, Moment 1 and Pseudotime, but higher values in Moment 5 and Sphericity in both SENS and MOT. (D) Multivariate analysis shows a significant influence of genotype on the analyzed outcome variables in the STR. Post-hoc analysis on univariate comparisons indicates that all variables, except for volume contributed to the rejection of H0. The KO leads to lower values in Surface Area, Volume Ratio, Moment 1 and Pseudotime, but higher values for Moment 5 and Sphericity. Overall effect sizes are smaller than those observed in cortical regions. In (A), semi-parametric MANOVA with pairwise multivariate Tukey post-hoc analysis was performed (sample sizes as number of synapses analyzed are 4084, 5171 and 4843 for STR, MOT and SENS, respectively). A univariate analysis with Bonferroni-corrected post-hoc analysis was added to identify variables contributing to the rejection of H0. Finally, p-values reported in the figure were calculated via pairwise Benjamini-Hochberg-corrected post-hoc analysis for each univariate analysis. In (B-D), semi-parametric MANOVA with univariate Bonferroni-corrected post-hoc analysis was performed to calculate p-values for all pairwise comparisons (sample sizes as number of synapses analyzed in WT-/KO-mice in B-D are 4843/5478, 5171/7493 and 4084/4219, respectively). Modified ANOVA-type statistics (MATS) and p-values representing the overall multivariate analysis are reported within the respective figure panels. The values shown have been centered and scaled. NS. $p > .01$, * $p \leq .01$, ** $p < .001$, *** $p < .0001$. Detailed statistical analysis, including the code to generate the plots is provided as R script in the supplementary materials.

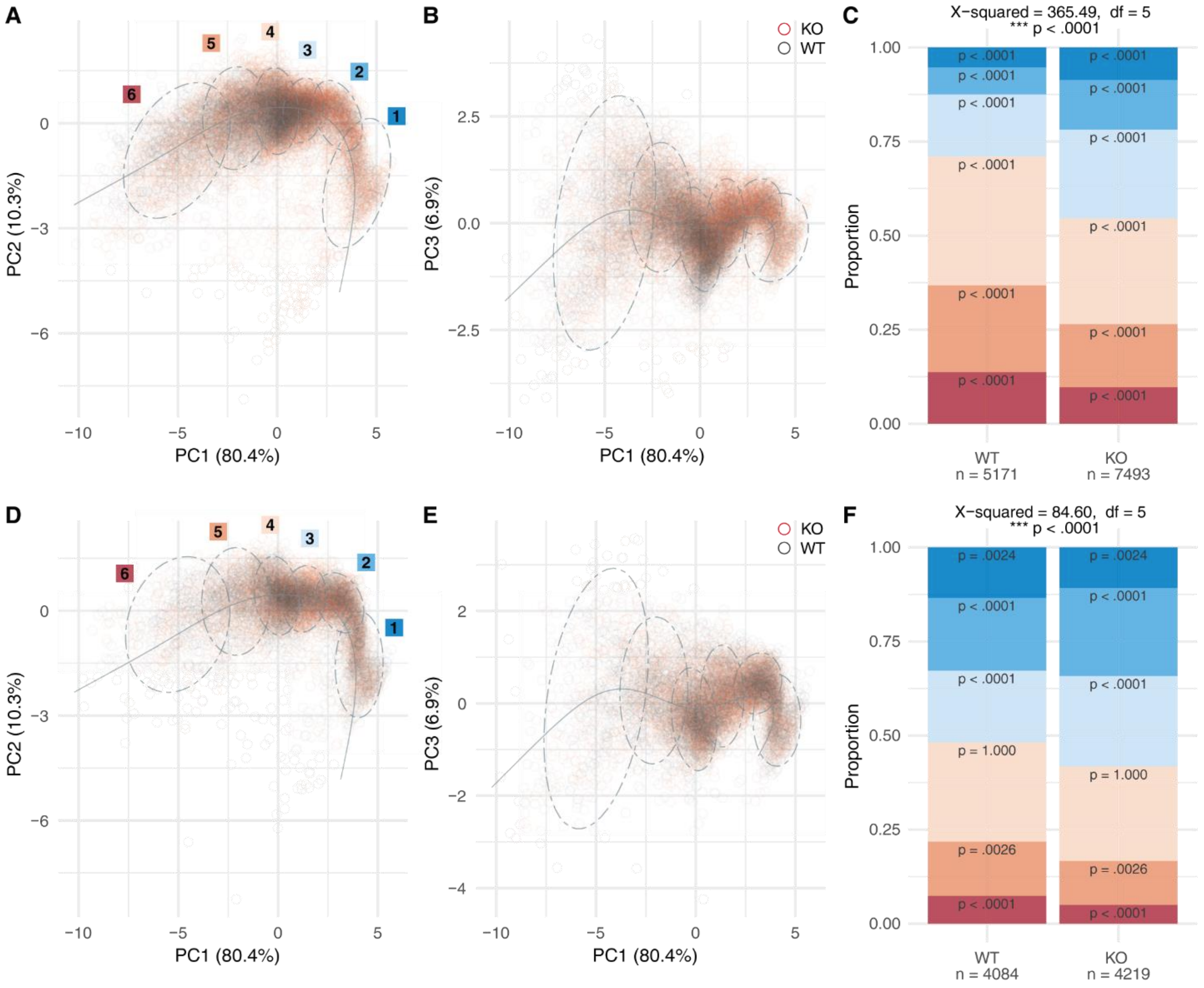

Fig. S7. Shape characteristics define genotype dependent synaptic fingerprint in the motor cortex & striatum.

(**A/B & D/E**) Scatterplots representing shapes of postsynaptic DLG4 scaffolds (one circle per shape) projected into a three-dimensional space defined by principal components (PC) 1-3. Shapes are partionally clustered via CLARA into six distinct categories as visualized by ellipsoids representing the 95% confidence level for a multivariate t-distribution. The fitted pseudotime curve is overlayed in each 2D scatterplot. Color-coding represents genotypes wild type (WT) and SHANK3-KO (KO) as indicated in the legend. Distributions in (A/B) and (D/E) are derived from

the motor cortex and striatum, respectively. Ellipsoids in (A & D) are labelled with the cluster identity. **(C/F)** Bar plots display the proportional distribution of clusters as visualized and color-coded in (A & D). (C) Chi-square test for independence reveals that genotype (WT vs. KO) does influence synapse cluster identity in the motor cortex, with all clusters deviating significantly from the expected proportions. SHANK3-KO mice presents with a clear overrepresentation of small/spherical clusters 1-3, while voluminous/complex shaped clusters 4-6 are less abundant when compared to WT animals, as evident in the scatterplots (A/B). (F) Chi-square test for independence reveals that genotype influences synapse cluster identity in the striatum less clearly and that all clusters deviated significantly from the expected proportions, except for cluster 4. KO leads to an overrepresentation of small/spherical clusters 2/3, while voluminous/complex shaped clusters 5/6 are less abundant when compared to WT. Noteably, cluster 1 seems to be more dominant in the WT-group. Differences are also not as readily visible in the scatterplots of (D/E). The respective sample size is indicated in (C & F). *** $p < .0001$. Detailed statistical analysis, including the code to generate the plots is provided as R script in the supplementary materials.

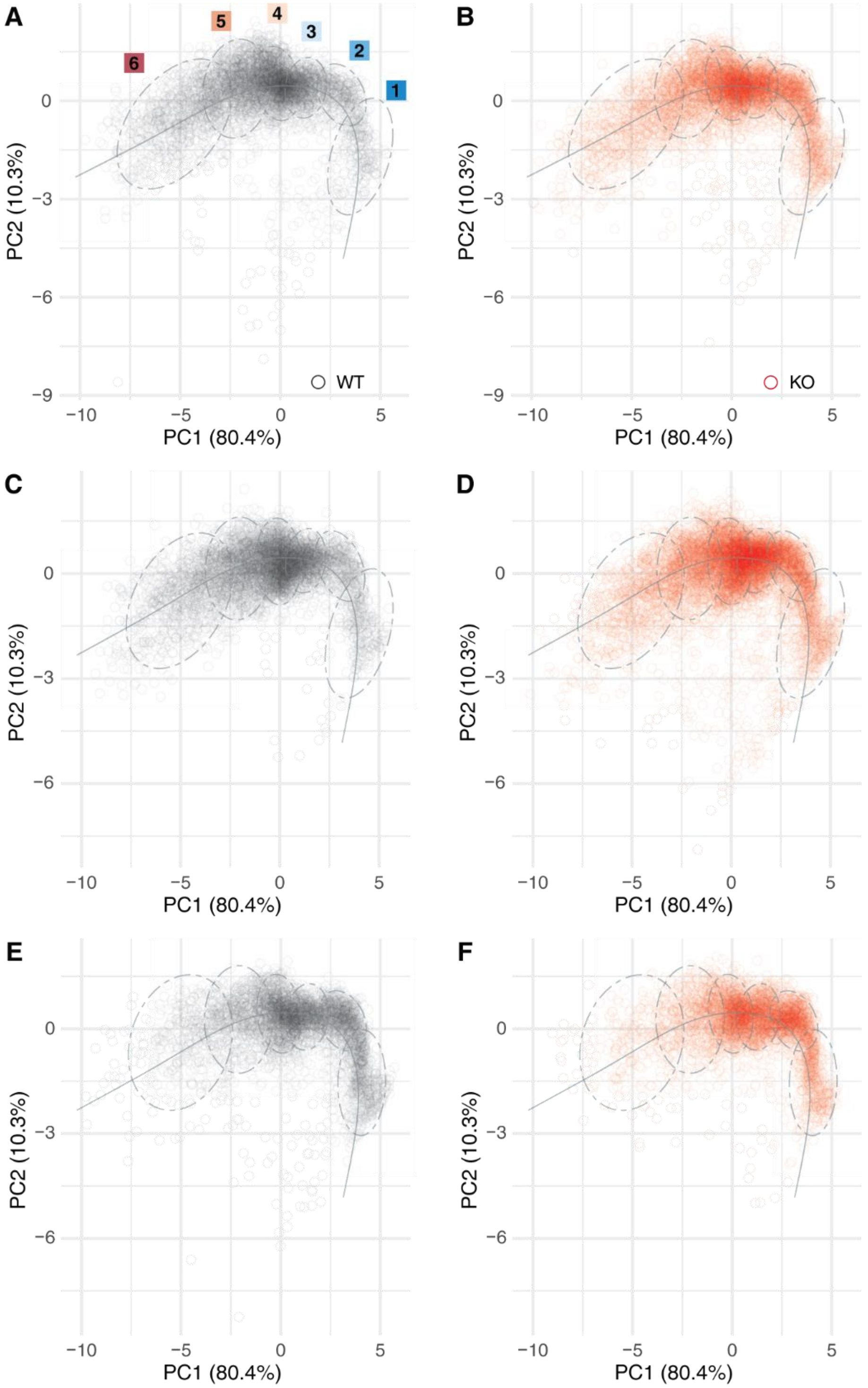

Fig. S8. Visual comparison of genotype dependent synaptic fingerprints across brain regions. (**A-F**) Scatterplots representing shapes of postsynaptic DLG4 scaffolds (one circle per shape) projected into a two-dimensional space defined by principal components (PC) 1 & 2. Shapes are

partionally clustered via CLARA into six distinct categories as visualized by ellipsoids representing the 95% confidence level for a multivariate t-distribution. The fitted pseudotime curve is overlayed in each 2D scatterplot. Color-coding represents genotypes wild type (WT, A/C/E) and SHANK3-KO (KO, B/D/F) as indicated in the legend in (A/B). Distributions in (A/B), (C/D) and (E/F) are derived from the sensory cortex, motor cortex and striatum, respectively. Ellipsoids in (A) are labelled with the cluster identity, which is consistent across all plots. The code to generate the plots is provided as R script in the supplementary materials.

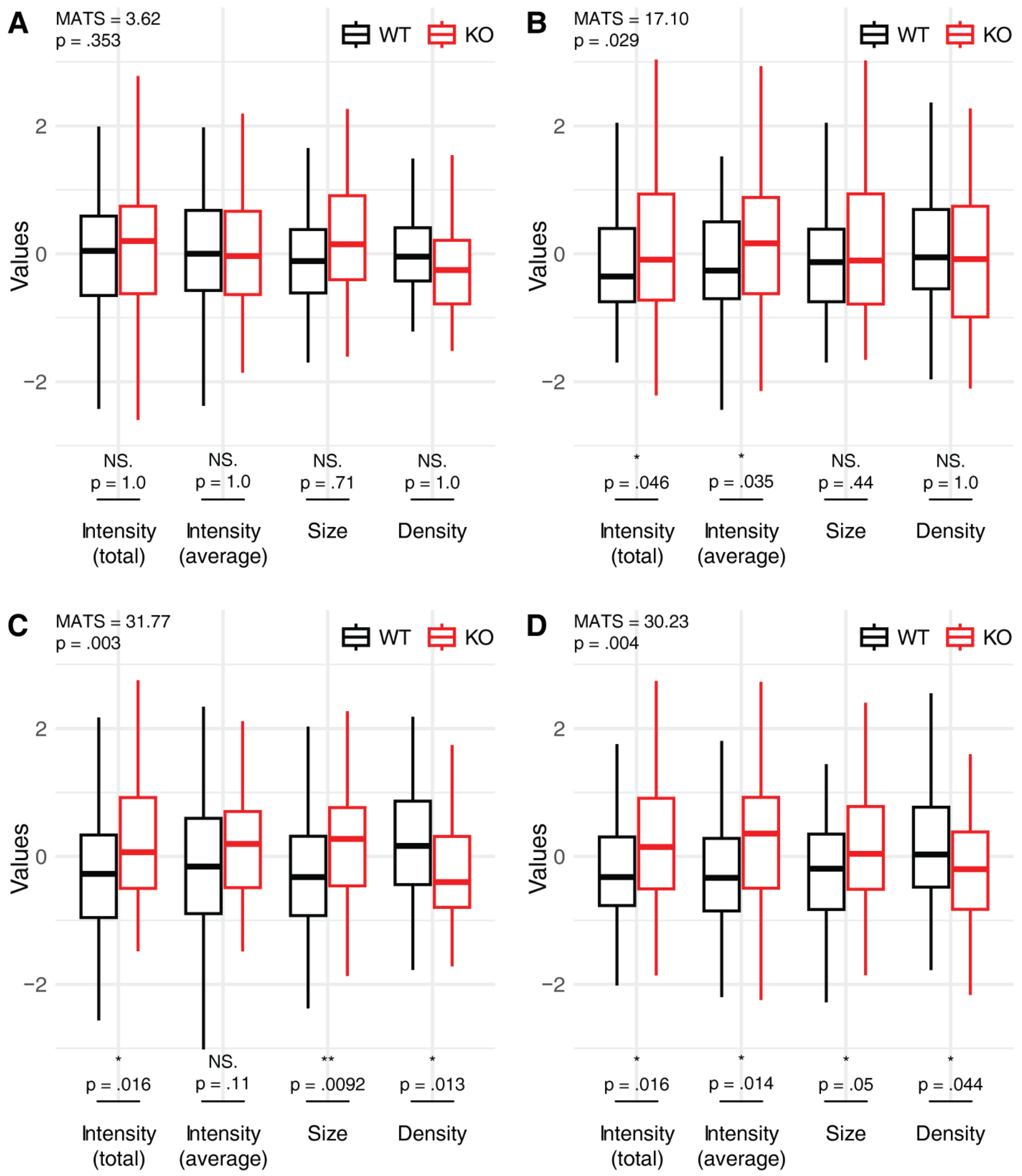

Fig. S9. Basic properties of subsynaptic organization vary across genotypes.

(**A-D**) Basic characterization of subsynaptic particles in the striatum and sensory cortex of wild type (WT) and SHANK3-KO (KO) mice, visualized by box plots. (A) HOMER1 particles remain unchanged across genotypes in the sensory cortex. (B) The analysis of DLG4 in the striatum reveals a subtle increase of intensity (total & average), similar to the sensory cortex, while size

and density are unaffected. (C/D) BSN shows consistent alterations in the sensory cortex (C) and striatum (D), with increased intensity and size of subsynaptic particles, but decreased density. The values shown have been centered and scaled. Semi-parametric MANOVA with univariate Bonferroni-corrected post-hoc analysis was performed to calculate the p-values for all pairwise comparisons. Modified ANOVA-type statistics (MATS) and overall p-values are reported within the respective figure panel. Sample sizes as number of synapses analyzed in WT-/KO-mice are 60/66 in (A), 125/130 in (B), 69/73 in (C) and 71/71 in (D). NS. $p > .05$, * $p \leq .05$, ** $p < .01$. Detailed statistical analysis, including the code to generate the plots is provided as R script in the supplementary materials.

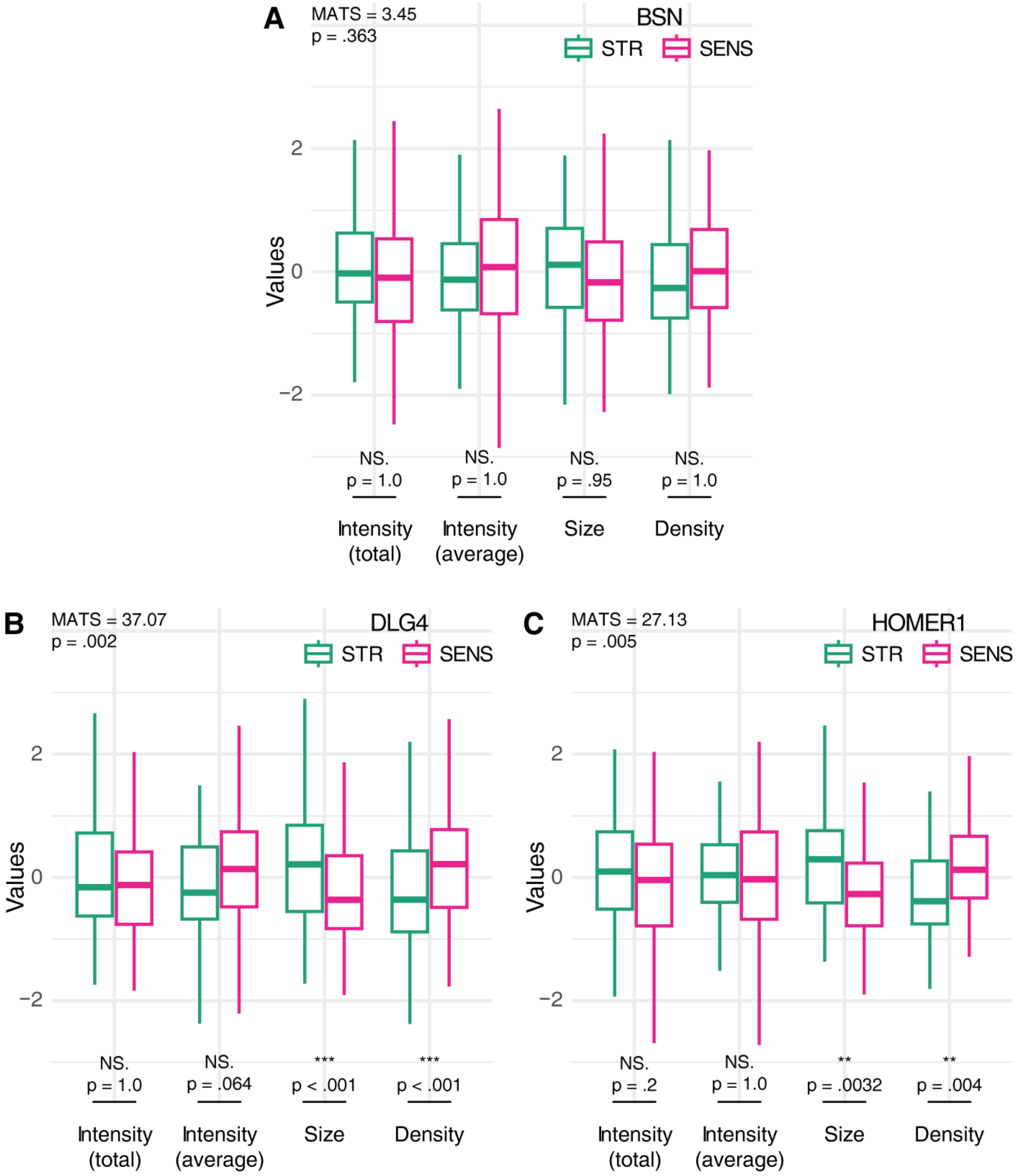

Fig. S10. Basic properties of subsynaptic organization vary across brain regions.

(**A-C**) Basic characterization of subsynaptic particles in the striatum (STR) and sensory cortex (SENS) of wild type mice, visualized by box plots. (A) BSN particles remain unchanged across brain regions. (B/C) The analysis of DLG4 and HOMER1 reveals a decreased size, but increased density of subsynaptic particles in the sensory cortex, when compared to the striatum. The values

shown have been centered and scaled. Semi-parametric MANOVA with univariate Bonferroni-corrected post-hoc analysis was performed to calculate the p-values for all pairwise comparisons. Modified ANOVA-type statistics (MATS) and overall p-values are reported within the respective figure panel. Sample sizes as number of synapses analyzed in STR/SENS are 71/69 in (A), 125/129 in (B), and 54/60 in (C). NS. $p > .05$, ** $p < .01$, *** $p < .001$. Detailed statistical analysis, including the code to generate the plots is provided as R script in the supplementary materials.

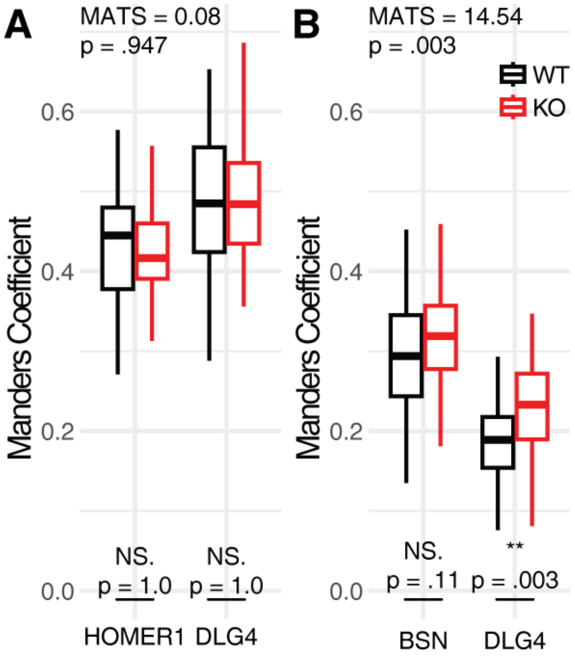

Fig. S11. Colocalization characteristics of subsynaptic particles across genotypes.

(**A-B**) Colocalization characteristics of subsynaptic particles in the striatum and sensory cortex of wild type (WT) and SHANK3-KO (KO) mice, visualized by box plots. (A) Correlation analysis via Manders Coefficients reveals that both from the perspective of HOMER1 and DLG4 colocalization is unchanged when WT and KO are compared in the sensory cortex. (B) However, from the perspective of striatal DLG4 particles, colocalization with presynaptic BSN was increased in the KO. Semi-parametric MANOVA with univariate Bonferroni-corrected post-hoc analysis was performed to calculate the p-values for all pairwise comparisons. Modified ANOVA-type statistics (MATS) and overall p-values are reported within the respective figure panels. Sample sizes as number of synapses analyzed in WT-/KO-mice are 60/66 in (A) and 71/71 in (B). NS. $p > .05$, ** $p < .01$. Detailed statistical analysis, including the code to generate the plots is provided as R script in the supplementary materials.

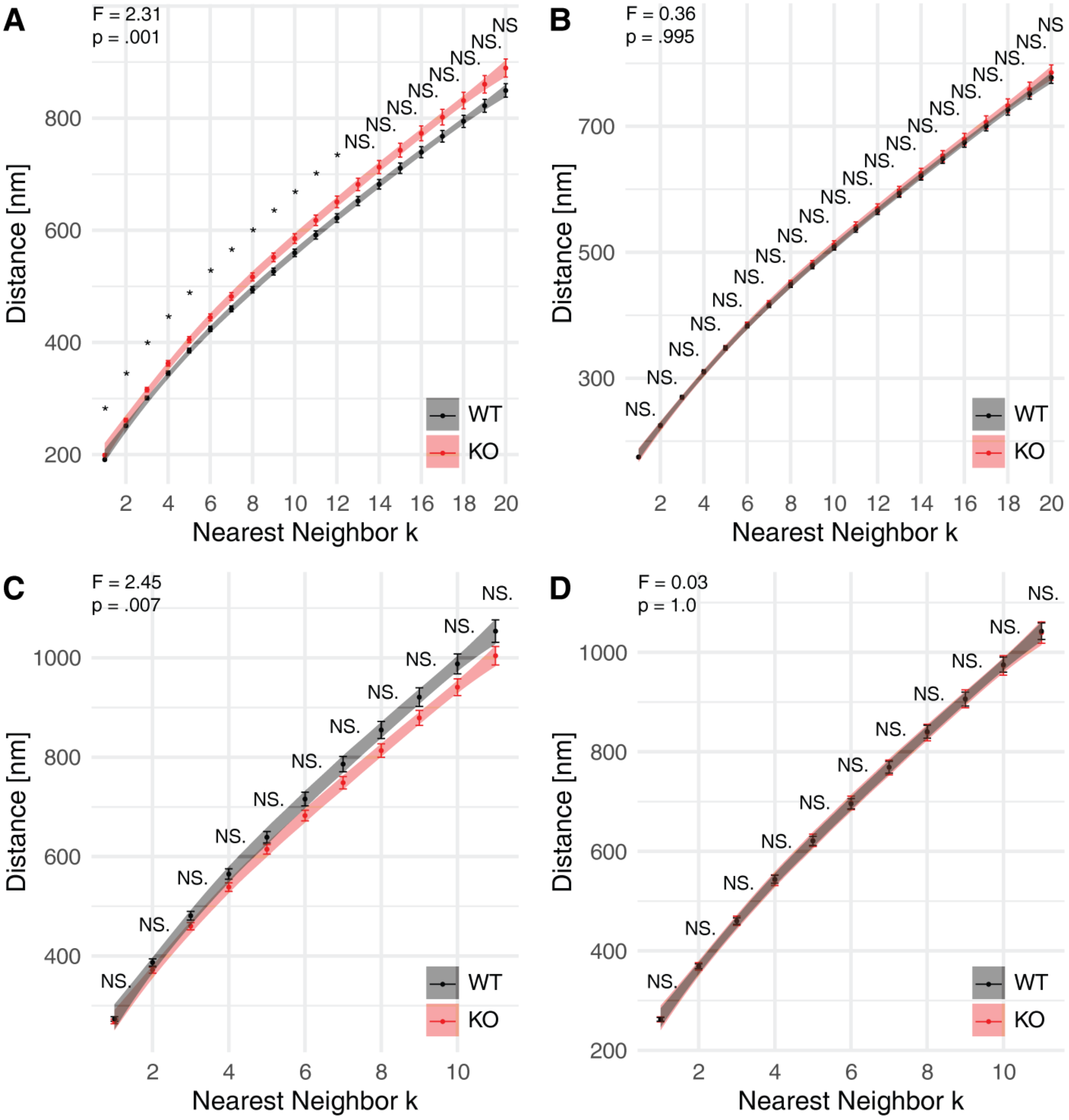

Fig. S12. Spatial pattern analysis via nearest neighbor distance (NND) of subsynaptic particles across genotypes.

(**A-D**) Nearest neighbor distance (NND) analysis of subsynaptic particles in the striatum and sensory cortex of wild type (WT) and SHANK3-KO (KO) mice, represented by point ranges showing the mean ±SEM per neighbor k and a fitted curve with its 95% confidence interval. (A) Spatial pattern analysis via NND suggests a reduced association of subsynaptic HOMER1 particles to their respective neighborhood in the sensory cortex since distances to neighbor k1 up to k12 are

increased. (B) However, NND analysis of striatal DLG4 particles shows no significant differences between genotypes. (C/D) Although overall ANOVA shows a significant result in the striatum (C), none of the pairwise post-hoc comparisons detects changes when subsynaptic BSN particles are analyzed in the striatum (C) and sensory cortex (D). Repeated measures ANOVA with Benjamini-Hochberg-corrected post-hoc analysis was performed for all pairwise comparisons. The F-value and overall p-values are reported within the respective figure panels. Sample sizes as number of synapses analyzed in WT-/KO-mice are 60/66 in (A), 125/130 in (B), 71/71 in (C) and 69/73 in (D). NS. $p > .05$, * $p \leq .05$, ** $p < .01$, *** $p < .001$. Detailed statistical analysis, including the code to generate the plots is provided as R script in the supplementary materials.

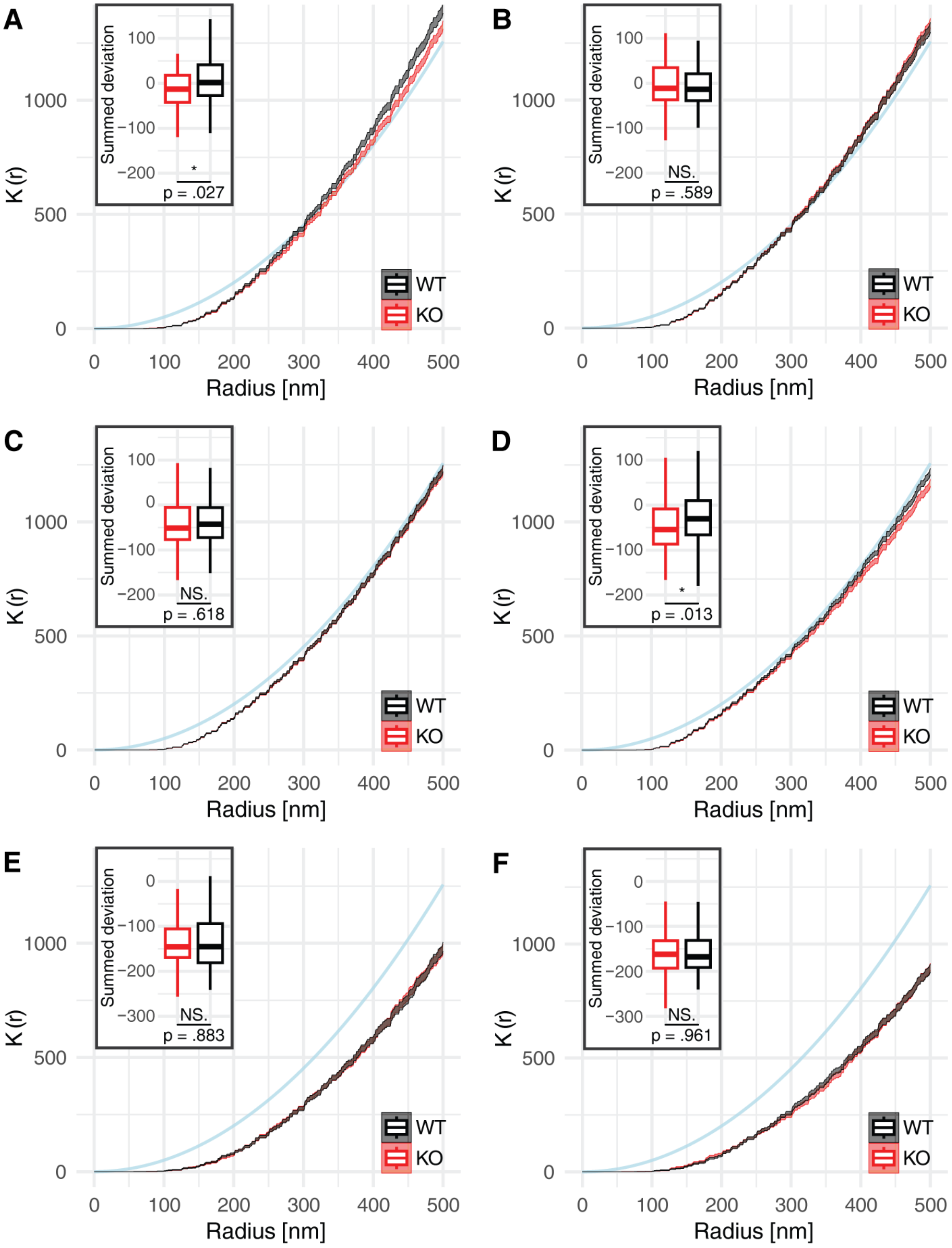

Fig. S13. Subsynaptic particle populations show unique spatial distributions and vary across genotypes.

(**A-F**) Averaged radial K-estimates of subsynaptic particles in the striatum (A & C & E) and sensory cortex (B & D & F) of wild type (WT) and SHANK3-KO (KO) mice as visualized by a fitted curve including its 95% confidence interval. A light blue reference line represents the estimate under the assumption of complete spatial randomness (CSR). Values above the reference indicate a clustered pattern, while values below indicate a more regular pattern than expected under CSR. Inlets in the upper left corner show the summed deviation from CSR per genotype. In both brain regions, HOMER1 exhibits a regular spatial point pattern at radial distances below 250 nm (A/B), but clustering tendencies above approximately 300 nm. This clustering tendency at high radial distances is pronounced in the WT when compared to SHANK3-KO in the striatum (A). No genotype effect can be observed in the sensory cortex (B). DLG4 also displays a regular particle distribution at radial distances below 200 nm in both striatal (C) and cortical (D) regions. At higher radial distances, K-values approach the CSR reference line with no apparent differences between genotypes in the striatum. However, SHANK3-KO mice exhibit a more regular patterning at radial distances above 400 nm in the sensory cortex. BSN exhibits a consistent regular distribution across all radial distances in both the striatum (E) and sensory cortex (F), suggesting a more general spatial patterning compared to HOMER1 or DLG4, however no apparent differences between genotypes are observable. Additional two-sample Wilcoxon tests reveal that the summed deviation from CSR is higher in the KO, when striatal HOMER1 or cortical DLG4 is analyzed, while all other conditions did not show significant changes. Sample sizes as number of synapses analyzed in WT-/KO-mice are 54/59 in (A), 60/66 in (B), 125/130 in (C), 129/139 in (D), 71/71 in (E) and 69/73 in (F). * $p \leq .05$. Detailed statistical analysis, including the code to generate the plots is provided as R script in the supplementary materials.

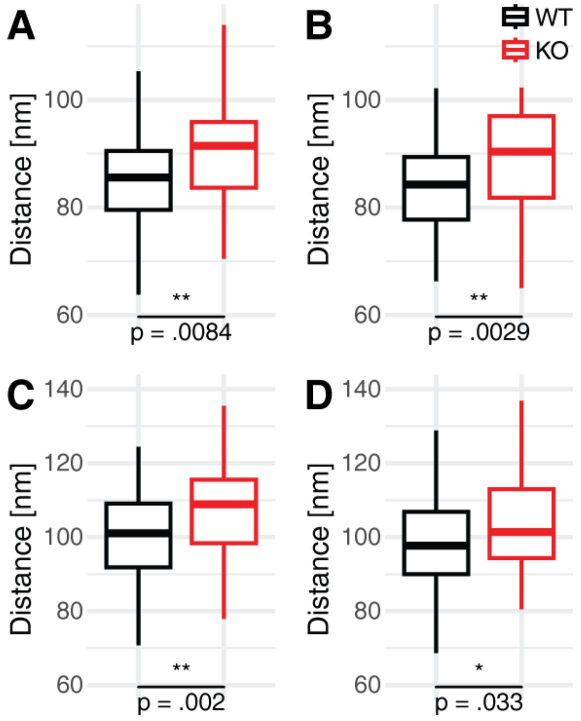

Fig. S14. Distance analysis of overlapping subsynaptic particles across genotypes.

(**A-D**) Centre of mass distance measurements of subsynaptic DLG4 particles overlapping with HOMER1 or BSN in the striatum or sensory cortex of WT and KO mice visualized by box plots. Two-sample Wilcoxon tests reveal that distances are significantly increased under SHANK3-deficient conditions in both the striatum (A & C) or sensory cortex (B & D), when either HOMER1-DLG4 (A/B) or BSN-DLG4 (C/D) particle pairs are analyzed. Sample sizes as number of synapses analyzed in WT-/KO-mice are 54/59 in (A), 60/66 in (B), 71/71 in (C) and 69/73 in (D). * $p \leq .05$, ** $p < .01$. Detailed statistical analysis, including the code to generate the plots is provided as R script in the supplementary materials.

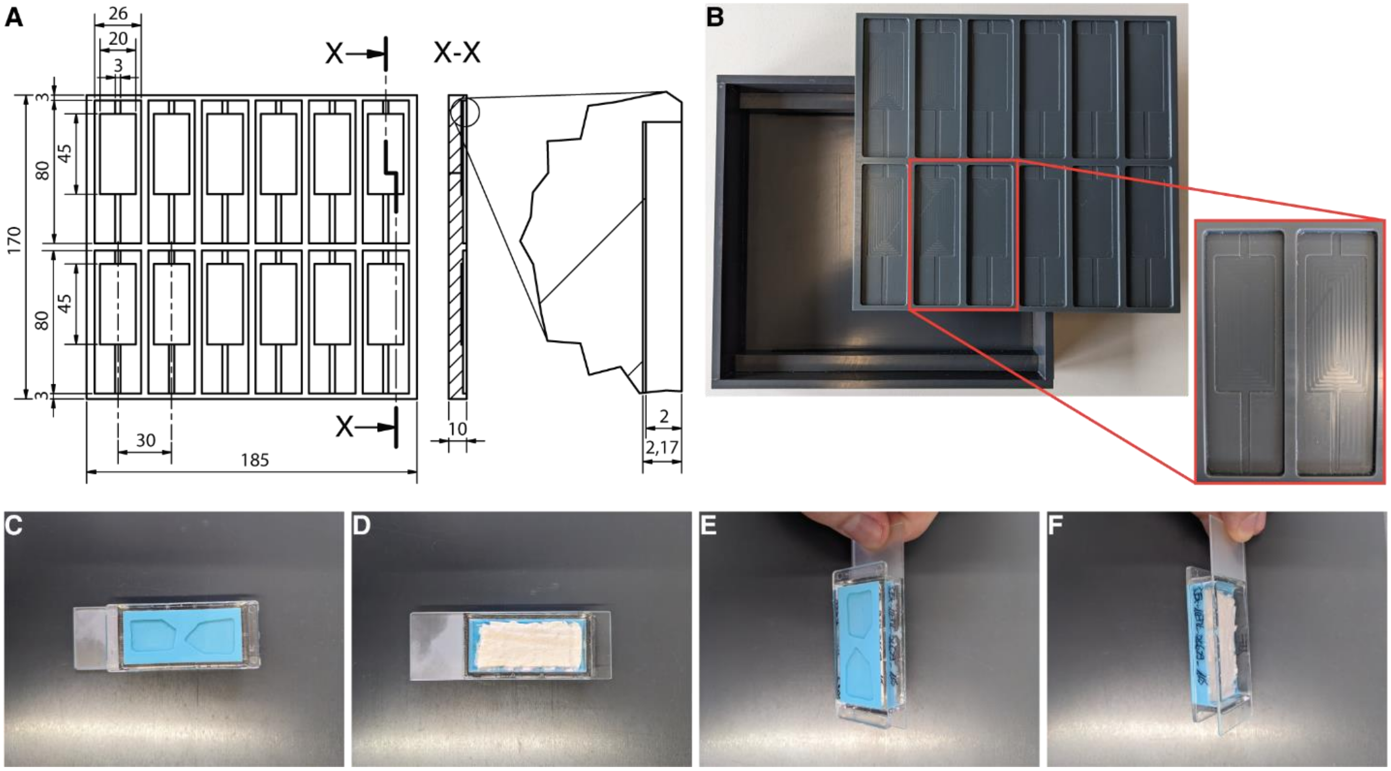

Fig. S15. Custom-built gelation chamber plate & imaging chamber.

(**A-B**) Illustrations showing the custom-built gelation chamber, which was used in this study. The gelation chamber was manufactured from polyvinyl chloride using a CNC machine (FP 2A, Deckel) and a custom design tailored to the needs of our standardized workflow. In (A) the technical drawing underlying the design realized by the CNC machine is shown. Notably, the wells are designed to fit standard microscope slides. During the gelation process the chosen well-depth results in a gel thickness of 170 µm. This is also visualized in the zoomed-in side view along the axis of the section defined by X-X. Measures of length are shown in millimeters. (B) shows pictures of the PVC-plate and the chamber, which can be filled with water and closed with a lid sealed by Parafilm M (Bemis) to maintain a humid environment during polymerization. (**C-F**) Pictures of two gels within an imaging chamber shown from various perspectives. Gels are embedded in picodent twinsil, topped by a wetted paper tissue, and finally sealed by a microscope slide fixed to the chamber via super glue.

Table S1. Usage of monomer solution batches across experiments

In this table, the used monomer solution batch is listed per associated figure or analysis.

Monomer solution batch	Associated figures & analysis	Mode of microscopy
A	Fig. 3, S2, S4-8	Confocal ExM
B	Fig. 4-5, S3, S9-14	ExM-STED

Table S2. Immunolabeling conditions

In this table, immunolabeling conditions are listed, including all primary and secondary antibodies (Ab) with their respective RRID, if available. All gels were additionally stained with an NHS ester dye coupled to BODIPY-FL to provide ultrastructural context during microscopy.

Figure	Target prim. Ab	Host	Dilution	RRID (prim. Ab)	Tag sec. Ab	Dilution	RRID (sec. Ab)
1B/C, 4A, S2B/BB, S3A/D	HOMER1	rb	1:200	SySy Cat# 160 022, RRID:AB_2619857	Abberior STAR ORANGE	1:200	Abberior Cat# STORANGE-1002-500UG, RRID:None
	DLG4	ms	1:200	SySy Cat# 124 011, RRID:AB_10804286	Abberior STAR RED	1:200	Abberior Cat# STRED-1001-500UG, RRID:None
2A/B & S2A/AA	DLG4	ms	1:200	SySy Cat# 124 011, RRID:AB_10804286	Abberior STAR ORANGE	1:200	Abberior Cat# STORANGE-1001-500UG, RRID:AB_2847853
	SHANK3	rb	1:250	Home-made, RRID: None	Abberior STAR RED	1:200	Abberior Cat# STRED-1002-500UG, RRID:AB_2833015

Figure	Target<br>prim. Ab	Host	Dilution	RRID (prim. Ab)	Tag<br>sec. Ab	Dilution	RRID (sec. Ab)
2C	SHANK3	rb	1:250	Home-made, RRID: None	Abberior STAR ORANGE	1:200	Abberior Cat# STORANGE-1002-500UG, RRID:None
	DLG4	ms	1:200	SySy Cat# 124 011, RRID:AB_10804286	Abberior STAR RED	1:200	Abberior Cat# STRED-1001-500UG, RRID:None
2D-F, 4E & S2C/CC	BSN	rb	1:200	SySy Cat# 141003, RRID:AB_887697	Abberior STAR ORANGE	1:200	Abberior Cat# STORANGE-1002-500UG, RRID:None
	DLG4	ms	1:200	SySy Cat# 124 011, RRID:AB_10804286	Abberior STAR RED	1:200	Abberior Cat# STRED-1001-500UG, RRID:None
3D / S2E&EE	DLG4	ms	1:200	SySy Cat# 124 011, RRID:AB_10804286	Abberior STAR ORANGE	1:200	Abberior Cat# STORANGE-1001-500UG, RRID:AB_2847853

Figure	Target prim. Ab	Host	Dilution	RRID (prim. Ab)	Tag sec. Ab	Dilution	RRID (sec. Ab)
	VGLUT1	gp	1:200	SySy Cat# 135 304, RRID:AB_887878	Abberior STAR RED	1:200	Abberior Cat# STRED-1006-500UG, RRID:None
S2D/DD & S3B/E	GRIA2	ms	1:200	SySy Cat# 182 111, RRID:AB_10645888	Abberior STAR ORANGE	1:200	Abberior Cat# STORANGE-1001-500UG, RRID:AB_2847853
	CAMK2A	rb	1:200	Abcam Cat# ab103840, RRID:AB_10900968	Abberior STAR RED	1:200	Abberior Cat# STRED-1002-500UG, RRID:AB_2833015
S2F/FF & S3C/F	HOMER3	rb	1:200	SySy Cat# 160 303, RRID:AB_10804288	Abberior STAR ORANGE	1:200	Abberior Cat# STORANGE-1002-500UG, RRID:None
	VGLUT1	gp	1:200	SySy Cat# 135 304, RRID:AB_887878	Abberior STAR RED	1:200	Abberior Cat# STRED-1006-500UG, RRID:None
